# Robotic Assisted Cannulation of Occluded Retinal Veins

**DOI:** 10.1371/journal.pone.0162037

**Published:** 2016-09-27

**Authors:** Marc D. de Smet, Thijs C. M. Meenink, Tom Janssens, Valerie Vanheukelom, Gerrit J. L. Naus, Maarten J. Beelen, Caroline Meers, Bart Jonckx, Jean-Marie Stassen

**Affiliations:** 1 MicroInvasive Ocular Surgery Center (MIOS sa), Lausanne, Switzerland; 2 Preceyes nv, Einhoven, The Netherlands; 3 ThromboGenics nv, Leuven, Belgium; 4 Medanex Clinic BVBA, Diest, Belgium; 5 Crystapharm BVBA, Lubbeek, Belgium; University of Tennessee, UNITED STATES

## Abstract

**Purpose:**

To develop a methodology for cannulating porcine retinal venules using a robotic assistive arm after inducing a retinal vein occlusion using the photosensitizer rose bengal.

**Methodology:**

Retinal vein occlusions proximal to the first vascular branch point were induced following intravenous injection of rose bengal by exposure to 532nm laser light delivered by slit-lamp or endolaser probe. Retinal veins were cannulated by positioning a glass catheter tip using a robotically controlled micromanipulator above venules with an outer diameter of 80μm or more and performing a preset piercing maneuver, controlled robotically. The ability of a balanced salt (BSS) solution to remove an occlusion by repeat distention of the retinal vein was also assessed.

**Results:**

Cannulation using the preset piercing program was successful in 9 of 9 eyes. Piercing using the micromanipulator under manual control was successful in only 24 of 52 attempts, with several attempts leading to double piercing. The best location for cannulation was directly proximal to the occlusion. Infusion of BSS did not result in the resolution of the occlusion.

**Conclusion:**

Cannulation of venules using a robotic microassistive arm can be achieved with consistency, provided the piercing is robotically driven. The model appears robust enough to allow testing of therapeutic strategies aimed at eliminating a retinal vein thrombus and its evolution over time.

## Introduction

To treat certain retinal vascular pathologies such as retinal vein occlusion (RVO), arterio-venous malformations or even retinal macroaneurysms, it would be useful to have a reliable means of cannulating the retinal vasculature. Among the retinal vascular pathologies, RVO is particularly common. RVO is the second most common cause of vision loss after diabetic retinopathy, with a prevalence of 5 per 1000 population [1,2]. Treatments for RVO have been directed at the vision threatening consequences, namely retinal neovascularization and macular edema. These have included observation, laser photocoagulation (ablative and subthreshold) [3,4], systemic thrombolysis, hemodilution [5], radial optic neurotomy [6,7], vitrectomy [8–10] and intravitreal injections of anti-vascular growth factors or sustained release preparations of steroids [11–15]. These modalities do not resolve the occlusion but act as temporizing measures, limiting the damage caused by the occlusion. A permanent solution for vascular occlusive disorders requires revascularization by either removing the occlusion pharmacologically or surgically [16], or by creating *de novo* anastomotic channels [17,18]. Cannulation of veins gives direct access to the clot, which is then amenable to treatment. There is some suggestion that the simple act of injecting saline or a balanced salt solution in a cannulated vein could dislodge a clot.

Unassisted cannulation of retinal veins is possible in cadaver eyes, as far as 15 disk diameters from the optic nerve [16,18,19]. In general, as the distance from the optic nerve increases, assistive devices are required to stabilize the vein or the cannula [16,18,19]. With increasing distance, visualization becomes more critical, prompting some to opt for endoscopy using GRIN lenses or to revert to an open sky approach in which the cornea and lens are removed [19]. In live surgery, cannulation has been limited to vessels located close to the optic nerve where a larger lumen and relative stability provided by adventitial fixation to the optic nerve facilitate the procedure [18,20,21]. To overcome some of these limitations, robotics has been proposed as a solution. Robotic assistance increases surgical accuracy 5- to 10-fold, while providing tremor damping and motion scaling [22–28]. Several authors have shown that catheterization of small veins is possible in cadaver eyes using a robotic system [25–28]. A decrease in vascular wall plasticity following death, as well as the presence of clotted blood further along the vascular path facilitates such procedures. Ueta *et al*. cannulated with success retinal veins in live cats in a laboratory setting [25]. No one has so far cannulated veins in an operating room set-up. Nor have veins proximal to a vascular obstruction been cannulated.

In the current paper, we used the Preceyes robotic micromanipulator to cannulate veins in anesthetized pigs following the induction of a retinal vein occlusion using a modification of the rose bengal methodology. We tested the feasibility of using a micromanipulator in a surgical setting, using standard operating room equipment. A comparison was made of the ease and success of cannulation with or without the use of a computerized piercing protocol. Once cannulated, the ability to dislodge the clot by perfusing the cannulated vein with a balanced salt solution was also assessed.

## Materials and Methods

### Animal use

All animals were housed and cared for in compliance with the FELASA guidelines and recommendations. The study was approved by the Ethics Committee for Animal Research at Medanex Clinic (EC MxCl-2013-017; Diest, Belgium) and conformed to the ARVO statement for the use of animals in ophthalmic and vision research.

Farm pigs of 5–7 weeks (Topig 20, cross-breeding of Yorkshire and Landrace pigs, Diest, Belgium) were induced with an intramuscular (IM) injection of 2.2 mg/kg xylazine (Xyl-M^®^ 2%; V.M.D.nv/sa, Arendonck, Belgium) and 4.4mg/kg tiletamine-zolazepam (Zoletil 100^®^, Virbac, Carros, France). Animals were intubated with a cuffed endotracheal tube (internal diameter of 6–7.5 mm) (Kruuse, Langeskov, Denmark). The animals were placed in prone position on the operating table, and connected to a mechanical ventilator. Anesthesia was maintained with 1.5%-2% isoflurane (IsoFlo^®^, Ecuphar, Oostkamp, Belgium) for the duration of the experiment. Mechanical ventilation was provided with a volume-controlled ventilator (Cicero; Dräger, Lübeck, Germany) at a tidal volume of 8–10 mL/kg body weight with an inspiratory oxygen fraction (FiO2) of 0.5.

A saturation probe was attached and a venous catheter was inserted in auricular vein for administration of fluids and drugs.

As per protocol, once anesthetized, the animals were positioned for the induction of a retinal vein occlusion (as described below). The presence of an occlusion was confirmed by OCT and fluorescein angiography (Spectralis HRA-OCT, Heidelberg Engineering, Heidelberg, Germany), performed about 30 minutes after induction of the vein occlusion. After confirming the presence of an occlusion, animals were transferred to an operating table where the cannulation procedure was performed. At the end of the experiment, the animals were either transferred to a recuperation pen for follow-up OCT and fluorescein angiography at day 19, or they were euthanized by an intravenous bolus of 20ml T61 (Intervet Int, Boxmeer, The Netherlands). In all cases, eyes were collected for histologic analysis. Eyes were obtained after the animals were euthanized.

Following an appropriate time in a recuperation pen, animals kept for follow-up were housed in groups of 3 to 7 pig, depending on body weight. Animals were kept on plastic grill floors with cage enrichment such as ad libitum access to drinking water. Cages were cleaned daily. Animals were inspected 2x/day by a care taker and once a week by a specialized veterinarian.

### Induction and confirmation of a retinal vein occlusion

To stabilize the pig eye, 10mL 2% Lidocaine was injected into the retroorbital space. Through an inguinal or antecubital vein, rose bengal 10 mg/kg (4,5,6,7-tetrachloro-2',4',5',7'-tetraiodo-fluorescein, Sigma-Aldrich, Diegem, Belgium) was injected intravenously. Two to seven minutes following the injection of rose bengal, a retinal vein was occluded proximal to its first branch point using an 532 nm laser (Oculight-GL532, Iridex, Mountain View, CA, USA) via a XL300 slit lamp (Takagi). The vasculature was visualized using a fundus laser lens (OGFA, Ocular Instruments, Bellevue, WA, USA). Laser settings were 120 mW, 75μm diameter, exposure 150 msec. Several applications were necessary along the intended vein, starting at the branch point and moving proximal along the vein. The laser was applied until there was evidence of occlusion (lack of flow) with up to 150 spots being applied. The minimal length of the occlusion corresponded to 3x the diameter of the vein.

In a subset of animals, the occlusion was induced by use of an endolaser, at the onset of the cannulation procedure. In these cases, there was no fluorescein confirmation of the presence of an occlusion. However, when the vein was cannulated, retrograde flow of BSS+ did confirm the presence of an occlusion. The laser settings were as follows: 140mW, exposure 110 msec.

Following laser treatment, in eyes with an occlusion generated via a slit lamp, the site of occlusion was examined by OCT, using a volume scan protocol using the “fast” preset in Automatic Real-Time mode, averaging 48 frames per image. Each volume covered 20°x25° and consisted of 31 B scans, 260 μm apart. A confocal scanning laser ophthalmoscope was used for fluorescein angiography using a standard 55° lens. For orientation, an infrared image was first acquired before intravenous injection of 1 ml of fluorescein (Fluorescein 10% Faure 0.5gr/mL, Novartis Pharma, Rotkreuz, Switzerland). Consecutive images were taken with late images taken 5 minutes after the fluorescein injection.

### The robotic system

**Fig 1:**
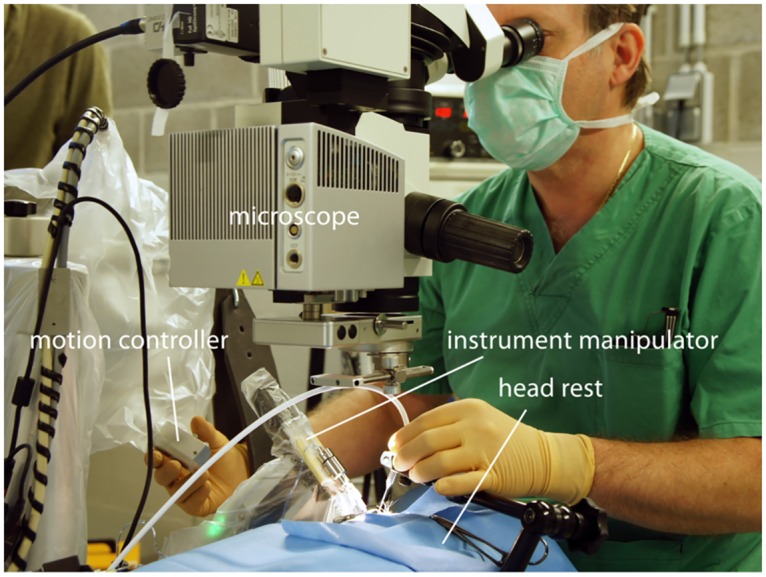
Surgical set-up used to operate the animals. The Preceyes micromanipulator is positioned for this intervention for the right eye of a pig. The motion controller is handled by the surgeon, from the temporal side of the eye within the surgical field and well within reach of the eye itself. The surgeon remains in his standard operating room position. In his left hand, he is holding a handheld endoilluminator (free hand).

The PRECEYES micromanipulator (PRECEYES, Eindhoven, the Netherlands) is a robotic assistive device designed to enhance surgical precision in vitreoretinal procedures. The system consists of a motion controller (MC), for hand motion input by the surgeon, and a table-mounted instrument manipulator (IM) holding the surgical instrument (Fig 1). When enabling the clutch on the MC, the coupling between the MC and the IM is activated. When coupled, the IM copies the movements that the surgeon makes using the MC, scaled as required for individual procedural step while filtering tremor using a programmed protocol. When the clutch is released, the IM holds a steady position, called the standby functionality. The IM is connected to a head rest, that is attached to a standard operating table. Alongside the MC is located a control cabinet, wrist band, touch screen and foot pedal. The surgeon can continue to monitor and guide the procedure via a regular ophthalmic microscope or other existing visualization system. Thanks to a compact design, the unit fits non intrusively at the head of the surgical table, allowing for hybrid surgery. The IM is only rotated into position when high precision tasks are required and kept out of the surgical field at other times.

**Fig 2:**
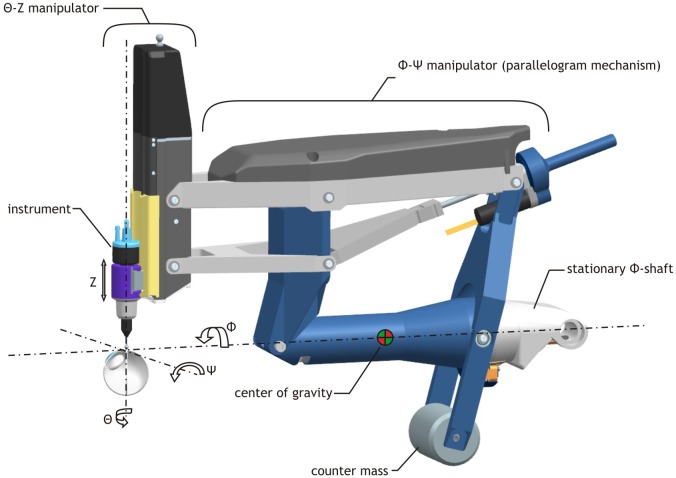
Design schematic of the micromanipulator. Shown are the the various axes of rotation and degrees of freedom (DOF). The parallelogram mechanism kinematically constrains the instrument pivot to the entry point into the eye.

The instrument manipulator has a serial layout, grouping the manipulated degrees of freedom (DoFs) into a Z-Θ manipulator and a Φ-Ψ manipulator. The Z-Θ manipulator relies on linear guides, ball bearings and rotary DC motors to be actuated. It is stacked to and manipulated along with the Φ-Ψ manipulator for which a double parallelogram mechanism was chosen. The parallelogram mechanism kinematically constrains the pivoting access point to the eye, while manipulating the instrument in Φ and Ψ directions (Fig 2). For overall safety, all DoFs are back driveable, allowing the surgeon or surgical assistant to overrule the actuator. To minimize the required actuator torques and to further improve inherent safety of the system, a counter weight is added, by which the center of gravity is brought to the Φ-axis. As a result, the weight of the Φ-Ψ manipulator is balanced when electronics or software fail, regardless of the orientation of the manipulator. The instrument manipulator weighs about 800 g, of which 480 g is contributed by the counter weight. With smartly designed actuators, equipped with high-resolution encoders, > 10 μm resolution is achieved at the instrument tip at the maximum insertion depth of 25 mm.

**Fig 3:**
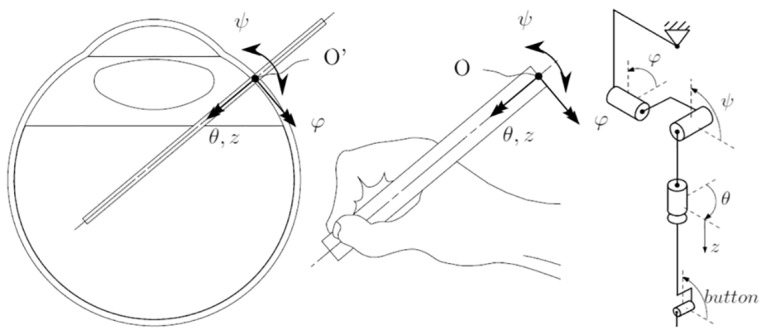
Design schematics of the motion controller. By placing the rotation point of the MC above his hand, a surgeon has the feeling of manipulating the tip of the instrument inside the eye.

The motion controller provides an ergonomic and intuitive working environment for the surgeon. Each instrument requires 4 degrees of freedom (DoFs) about the entry point and is operated by an additional DoF, e.g.: opening and closing of a forceps. A dedicated and compact, stylus-based 5-DoF haptic interface is provided. Having less mechanical links and pivot points than other 6 and 7-DoF systems, the system possesses a higher stiffness ensuring better performance. An intuitive working environment is created for the surgeon by placing the point of motion of his hand inside the eye. This is achieved by placing the rotation point of the MC above the surgeon’s wrist corresponding to the entry point of the instrument into the eye (Fig 3).

### Cannulation procedure

Following induction of a retinal vein occlusion as indicated above and its confirmation by fluorescein angiography, the animal was transferred to the operating room table, and positioned laterally for eye surgery. The eye was prepared and draped in standard surgical fashion. About 1 hour after the induction of the occlusion, three 25 gauge trocars were placed 2 mm posterior to the limbus. In the first 3 animals, a core vitrectomy (Dutch Ophthalmics ACS2500 Dual; Zuidland, the Netherlands) was carried out at the beginning of surgery. In the remaining animals, the eye was immediately prepared for vein cannulation as described below.

A large temporal sclerotomy was created with an MVR blade to a 1 mm outer diameter, allowing the insertion of a custom made trocar. This trocar allows for a precise positional alignment of the IM with the eye. After achieving a snug fit between the inner funnel of the trocar and the needle holder on the IM, a glass pipette with a terminal 30μm outer diameter and a beveled tip (Clunbury Scientific, LLC, Bloomfield Hills (MI), USA) was carefully advanced until visible in the vitreous cavity through the operating microscope. Illumination was provided by a standard 25G handheld light pipe (Dutch Ophthalmics ACS2500 Dual; Geervliet, the Netherlands), held at an appropriate distance to provide optimal illumination of the chosen cannulation site.

Under direct visualization, the surgeon could by means of the MC manipulate the needle and position the tip precisely over the vein to be cannulated. Several strategies to cannulate the veins were attempted. The most successful approach consisted in positioning the tip of the needle over the middle section of the vein, providing a slight indentation of the vein’s surface, then by means of an actuator, the needle was allowed to make a robotically controlled piercing motion through the wall of the vein. BSS+ was then injected into the vein via a variable speed syringe pump set on 14 μL/min (Model R99-E, Razel Scientific Instruments, Saint Albans, Vermont, USA) to confirm positioning. With retrograde flow, the venular system turned transparent, thus confirming cannulation and also the presence of a vein occlusion. The needle was then removed in retrograde fashion from the eye. In most cases removal of the needle did not cause any bleeding at the cannulation site. In non vitrectomized eyes, if bleeding occurred it was limited to a small localized hemorrhagic bleb.

### Histology

Eyes were obtained immediately after the animals were euthanized as described above. The eyes were fixed for 48 hours in 4% paraformaldehyde solution, rinsed in PBS before overnight storage in 3% sucrose in PBS. Eyes were slowly dehydrated in increasing ethanol gradient and xylol baths over a period of 3 weeks before paraffinization using a Shandon Excelsior ES tissue processor (Thermo Scientific, Ghent, Belgium). Paraffin embedded tissue blocks were sliced into 7μm sections. Selected sections were deparaffinized in a decreasing series of xylol and ethanol baths before staining the section with Hematoxilin and Eosin. Image acquisition was performed on a Zeiss Imager Z1 microscope, both under normal white light and using epifluorescence in the green channel. The latter was used to assess the presence of fibrin within the thrombus as described by Lucas TC et al [29].

## Results

**Fig 4:**
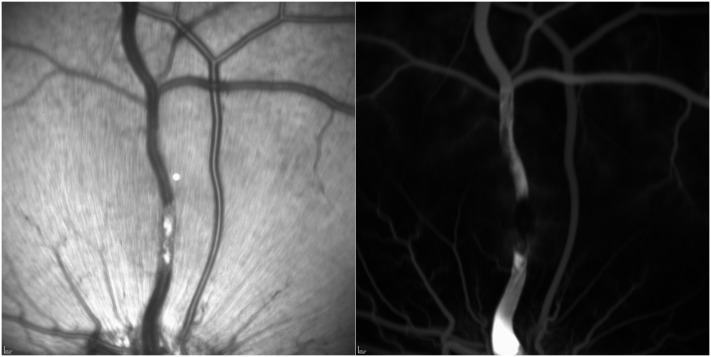
SLO image (left) and late frame fluorescein angiogram (right) showing an appropriate vein occlusion shortly after laser application. An occlusion measuring 3 vein diameters was generated without the development of retinal damage. The laser energy was limited to the lumen of the vessel. A late frame taken from the fluorescein angiogram shows blockage of fluorescein outflow through the occluded vein and retrograde filling from the optic nerve.

**Fig 5:**
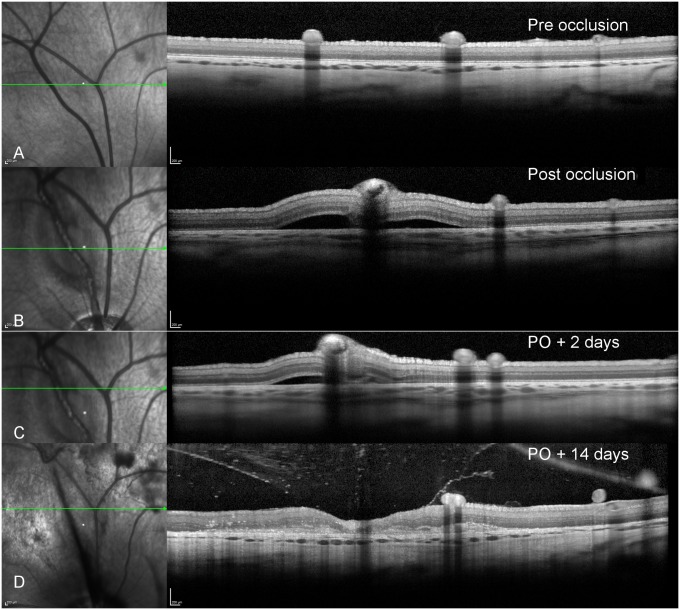
SLO image (left) and corresponding OCT image of the retina pre and post induction of a vein occlusion. OCT showing the site of a venous occlusion. A- prior to the induction of a thrombus. B- shortly after the creation of a venous occlusion, the outer diameter of the vein increases in size. An area of hyperfluorescence appears in the upper portion of the vascular lumen corresponding to the area of fibrin deposition within the thrombus (see Fig 7). In the lower portion, the area of hypofluorescence corresponds to a dense meshwork of erythrocytes, platelets and inflammatory cells. An associated serous detachment is also present, often appearing within 30 minutes of the induction of the occlusion. In panel C, 14 days after the induction of the thrombus, there is still retinal inflammation present as witnessed by hyper reflective dots in the retina and the vitreous. A partial posterior vitreous detachment is present containing a number of inflammatory cells and residual debris. The area around the vein is thinned as the outer retinal layers are reduced in size.

**Fig 6:**
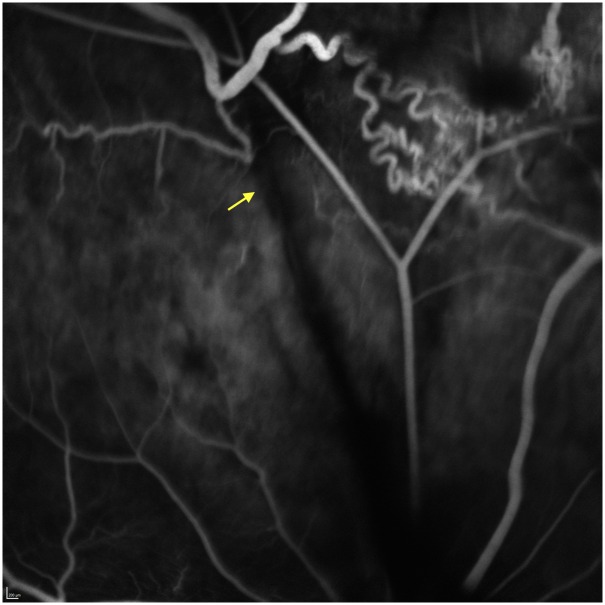
Day 14 fluorescein angiogram showing the presence of collateral vessels. Day 14 following a rose bengal induced venous occlusion in a pig eye. The formation of collateral vessels adjacent to the site of occlusion is clearly evident in this late frame angiogram. The site of occlusion is indicated by the arrow.

Using the proposed setting for laser intensity, an occlusion could be created in all cases, and was confirmed by angiography (Fig 4) and by OCT (Fig 5). When the length of vein treated was increased beyond 6 vein diameters, a serosanguinous detachment occurred within a few hours in most treated eyes. With practice it was possible to avoid damaging the neighbouring retina, concentrating the energy within the vascular lumen. However, this proved difficult to insure in all cases when using the slit lamp mode of delivery. Use of an endolaser probe lead to the formation of a more controlled occlusion with no collateral damage, as the laser could be more precisely targeted to the lumen of the vein. Within 72 hours, all eyes developed serous or sero-sanguinous retinal detachments associated with the formation of fibrin strands in the vitreous and inflammatory vascular sheathing which persisted to day 12 but resolved in all cases spontaneously by day 19. In some cases, a network of collateral vessels formed beyond the zone of occlusion prior to the resolution of the occlusion (Fig 6).

**Fig 7:**
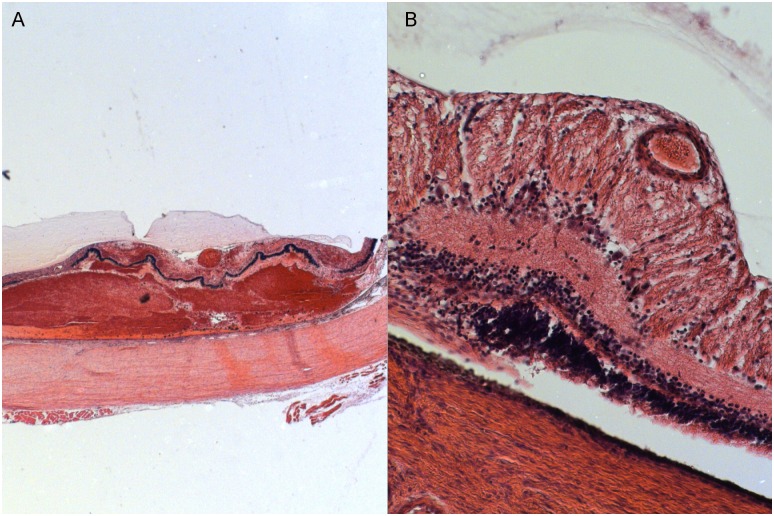
Hematoxylin-eosin stain (A-5x, B-10X) of an occluded vessel. Damage to the surrounding retina and underlying choroid is clearly visible following laser extending beyond the limits of the vascular wall. However, the vascular lumen is occluded with limited damage to the vessel wall. Extensive choroidal hemorrhage indicates that considerable damage was also induced in the choroidal circulation.

**Fig 8:**
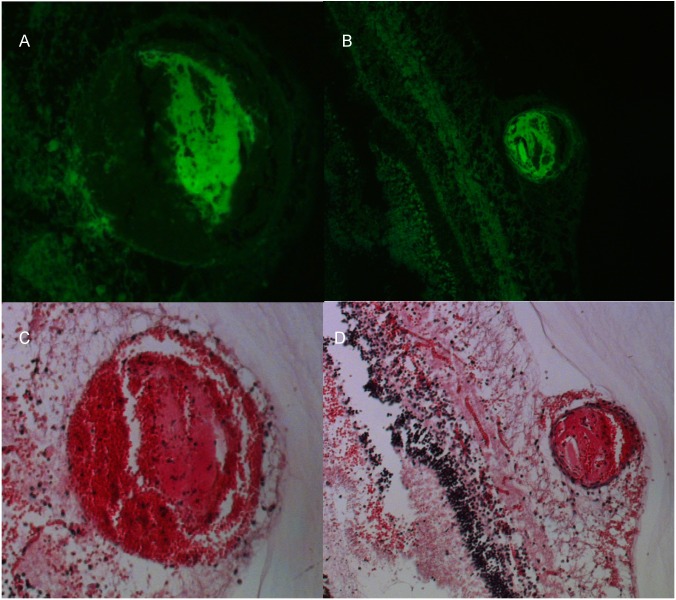
Green epifluorescence (A 20X, B 10X) and H&E stain (C: 20X, D:10X) of a laser induced vascular occlusion. The eye was collected within 2 hours of the occlusion. Epifluorescence in the green channel confirms the presence of fibrin while the remainder of the thrombus is composed of a network of platelets and erythrocytes and inflammatory cells.

On histology, following an intense laser barrage involving the paravascular retina, extensive collateral damage was observed to the retina and choroid. Hemorrhage was observed in the retina, the subretinal space and choroid. Interestingly, even under these conditions, the walls of the retinal vein remained intact, though thinning of the lamina propria was observed in heavily lasered sections (Fig 7). When the laser energy was limited to the lumen of the vein, the occlusion was characterized by limited damage to the vascular wall. The thrombus contained layers of fibrin (best seen in green epifluorescence) adjacent to a cellular component rich in erythrocytes and white blood cells (Fig 8).

Cannulation is best achieved shortly after the induction of the vein occlusion before the development of a serous retinal detachment. Attempts to cannulate over a detached area were made more difficult by the absence of posterior support as the needle was advanced. Blood present within the lumen of a vein can be easily displaced distally as pressure is exerted on the surface of the vein by the catheter. A problem which has not been well described by previous authors working on live animals. Several catheterization sites were tested, including direct venular cannulation along the free length of a vessel, vascular branching points, arterio-venous crossings, and an insertion proximal to the site of venous occlusion or within the clot itself. The most successful locations were either associated with some tethering of the vessel to underlying structures (vascular branch points or arterio-venous crossings) or were adjacent to the occlusion. Positioning the catheter directly into the clot is possible without much difficulty. Cannulation of a non tethered vessel proved difficult, unless some temporary pressure was placed distal to the proposed point of insertion to prevent egress of fluid.

Different piercing strategies were tested including side piercing which provided the best visualization of the catheter tip, but this motion is unnatural when using a robotic arm, and difficult to automate or control. Out of 6 penetration attempts, it was only successful once (17%). For this reason, this approach was abandoned.

An approach above the center of the vein using the protocol described in the methodology section gave the best results with all eyes being cannulated (9 eyes), when using an automated protocol designed to facilitate piercing of veins. Veins between 300 μm and 80 μm were cannulated using this combination. Manual cannulation using the motion controller without the piercing protocol were much less successful. Under these conditions, piercing through the superficial vessel wall, was frequently followed by a piercing motion through the inferior vessel wall (double piercing). Cannulation was successful in only 48% of such cases (24 of 52 attempts).

The angle of approach was also varied from direct vertical to an angle of 30° from vertical. Provided the tip and the indent on the surface of the vessel could be seen, the angle of approach did not seem to matter when using the automated piercing protocol. For unassisted manual cannulation, it is best to have a sufficient angle to clearly visualize the tip. The tip is advanced along the length of the vein attempting to create a mound of tissue ahead of the needle tip followed by a rapid advance through the venous wall.

**Fig 9:**
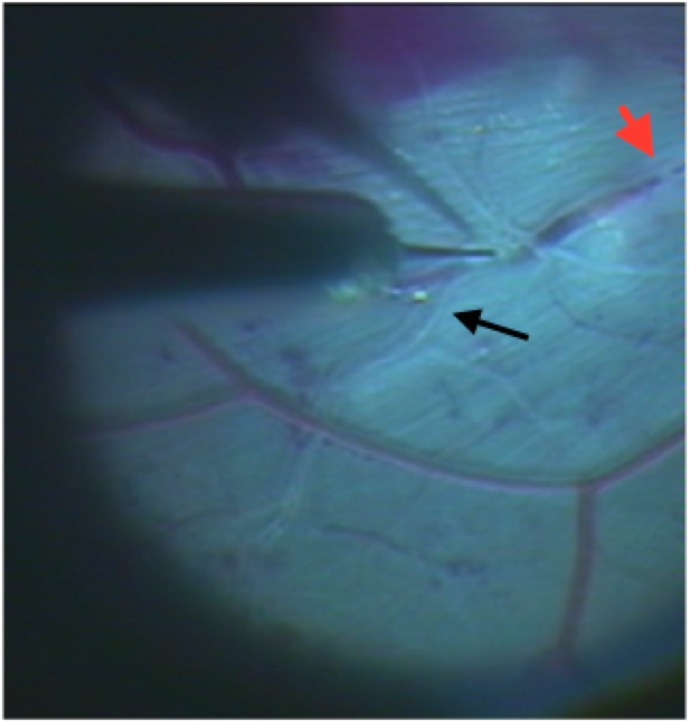
Retrograde filling of the retinal veins with BSS+ proximal to a vein occlusion. The black arrow shows the retrograde fill, while the red arrowhead the location of the occlusion.

A successful cannulation could be confirmed by the injection of BSS+ into the vein in 9 of 9 eyes cannulated using the piercing protocol. It lead to a retrograde filling of the venular system (Fig 9). When the infusion was stopped, the veins progressively refilled with blood. Re-opening of the infusion lead to refilling of the venular bed with transparent BSS+. If the site of injection was adjacent to the venous occlusion, all vessels to the edge of the clot were filled with fluid. Despite several cycles of BSS+ infusion, none of the clots were dislodged. If a more proximal location was chosen, particularly if beyond a venous bifurcation, the blood column adjacent to the clot remained intact, hence the BSS+ did not reach the location of the clot. Following successful cannulation, the catheter could remain in place for a prolonged period. We effectively left a venule cannulated for up to 20 minutes. However, upon stopping the infusion, the perfused vessel became less distended. As the vessel wall collapsed, the tip of the catheter would occasionally fall out of the vascular lumen. This did not happen if shortly after initiating the infusion of fluid, the catheter was advanced slightly within the lumen of the vessel.

## Discussion

There are four main challenges associated with retinal vein cannulation: (a) guiding the catheter tip accurately to the surface of the vein, (b) maintaining a sufficient blood column within the lumen of the vein, (c) piercing the vein wall and stopping the cannula insertion at the correct depth, (d) maintaining the tip stable during a subsequent drug injection. Several solutions have been proposed to overcome the guidance issue including handheld [30], teleoperated [31], and cooperatively controlled robotic devices [32]. All of these are able to resolve the issue of physiologic tremor (100μm) [33] relative to the size of the vessel to be cannulated (80μm) in *ex vivo* or the chick allantoic membrane model. As indicated by Becker *et al*, in addition to positional precision, and tremor filtering, the eye presents several constrains on motion, orientation and the explicit need to prevent collateral damage [31]. In principle these requirements can be provided by real time eye tracking, but eye motion can rapidly invalidate vision tracking algorithms, particularly at higher magnification when an instrument inserted through a trocar is moved within the eye. Another approach is to minimize eye motion by stabilizing the point of insertion and using it as a point of rotation into the eye. The same insertion point can be used as a reference point for all displacements within the vitreous cavity. Using this approach, it is possible to scale motions in such a way as to allow rapid translation through the vitreous, while providing slower positional precision as one approaches the retinal surface. An absolute motion stop set at the retinal surface, prevents inadvertent damage. Using this strategy, a surgeon can use an existing surgical microscope, while controlling and guiding a robotic arm to the required position on a retinal vein. Adequate illumination is provided by either a chandelier lighting system or a handheld light pipe. The latter approach is preferred to allow fine positioning of the catheter tip on the surface of the vein, as at higher magnification it may be difficult to clearly see a transparent glass tip. Here, shadows of the catheter in relation to the vessel surface when viewed from different angles is helpful. Modifying the surface of the catheter to enhance its visibility can also facilitate positioning [31].

Several groups have described the successful cannulation of venules in fresh cadaver eyes [18,19,23,26,34–36]. Cannulation is most easily accomplished close to the optic nerve, but is also possible several disk diameters away. Comparing live and cadaver eyes, the latter have less pliable vascular walls and a stable lumen making cannulation easier. In live animals, retinal veins tend to collapse upon light compression as the blood column is easily displaced towards the optic nerve. This adds to the challenge of a successful cannulation. When performing a peripheral venipuncture on vein located on the dorsal surface of a hand, a tourniquet is generally applied to maintain an appropriate blood volume. Large perceptible forces are applied which enable the clinician to sense a 10–20% difference in tissue resistance at the moment the needle enters the blood vessel [37]. To compensate for the lack of a tourniquet, it is necessary to make use of anatomical features that help stabilize the blood column, these include the passage of a vein over an artery, a branch point in the venous bed, or to insert the needle proximal to the occlusion itself. The latter provides the most stable blood column and is the easiest site for cannulation, followed by a branch point in the vascular bed. Cannulating a vein prior to its passage over an artery is less successful, unless some light pressure is applied on the distal vein to prevent egress of fluid. Positioning over the center of the vein is the most effective approach, similar to peripheral venipuncture [38]. Short beveled needles with a short angular approach also facilitate cannulation [32,39]. These two adaptations facilitate visualization and piercing, but the latter brings its own challenges and risks. Initial attempts were complicated by frequent double piercing through the outer wall. Compression of the vessel outer wall and a slow advancement allows a build up of pressure leading to the initial successful piercing action. However, once through the vessel, one needs to move from position control to force control otherwise the vessel recoil can lead to double piercing [37]. One solution to this problem is to have a forced sensing microneedle that can adjust the force as soon as a vessel is pierced [30]. Another is to develop an algorithm that takes into account vessel dimension and compliance at the time of the initial piercing action. This latter strategy was successfully implemented in our system. Once cannulated, the vessel remained cannulated for several minutes allowing for the infusion of balanced salt solution into the vascular network.

Parallel to our study of retinal vein cannulation, and indeed in the hope that we could reliably and reproducibly cannulate retinal veins, we were interested in establishing a model of retinal vein occlusion that could be immediately used in a surgical setting. Hence, animal morbidity would minimized, and the experimental conditions better controlled. In addition, we wanted to work with an animal whose ocular size and retinal architecture were similar to a human eye. Thus allowing us to simulate conditions encountered in human surgery. The porcine eye is in this respect an ideal model. Rose bengal, is a commonly used photosensitizer to induce vein occlusions but it causes a significant inflammatory response as a result of the free radicals that are generated upon exposure to the laser light. Reducing the length of vessel being treated and by using a small focussed laser beam limited to the diameter of the vessel, it is possible to limit collateral damage. Confirmation of an occlusion was obtained by fluorescein angiography, but as shown in Fig 5, the thrombus in an occluded retinal vessel has a unique OCT signature similar to its appearance in histology. Hence, OCT alone is sufficient to confirm the presence of a clot prior to any pharmacologic manipulation, avoiding fluorescein angiography and reducing the number of required manipulations in an anesthetized animal. The histology observed in a fresh thrombus revealed a clot composed of fibrin and an admixture of platelets and red blood cells. This appearance is identical to thrombi in central and branch retinal venous occlusions as reported in humans [40–42]. The reported degree of retinal edema, and subretinal hemorrhage correspond quite closely to our observations when laser damage to surrounding tissue is limited. Inflammation, a frequent component of vein occlusions appears in our case at about 72 hours, while in the cases studied by Green *et al*., inflammation was observed histologically in specimens older than 2 weeks. The difference may be due to the manner by which the occlusion is created, namely the use of rose bengal, a photo-sensitizer that produces free radicals, or possibly to the use of young age animals—capable of mounting a much more intense inflammatory response to a vascular insult than the typical patient in whom an occlusion occurs. Vascular sheathing though rare in older individuals, is not infrequently observed in young adults developing a vein occlusion [43].

We observed that the occlusion is of limited duration as most animals recanalize the occluded vessel within about 3 weeks. Similar results were obtained in rabbits and other models following rose bengal induced retinal vein occlusion [17,44–46]. Green *et al*. noted that small recanalization channels could be observed in human thrombi starting about 2 weeks after the initiating event. Over time, these smaller channels coalesce to form larger channels, a process that evolves over 1 or more years. Therefore, it will be possible to study with this model therapeutic responses to acute clots and with a delay of 1 to 2 weeks, the response of more mature clots to surgical/pharmacologic intervention. With the current experiments, it is clear that simple infusion of BSS+ and the dilation of the vein proximal to the occlusion is insufficient to lead to the displacement of the clot. With time, we noted the appearance of collateral anastomotic channels as has been previously described [46,47].

In conclusion, we describe a reproducible model of retinal vein occlusion in pigs that simulates branch retinal vein occlusions in humans. We further describe a reliable method of retinal vein cannulation by robotic (micromanipulator) assistance that can be carried out using existing surgical equipment and instrumentation. The robotic arm has inherent design features that are used to overcome the limitations associated with the cannulation of small venules at forces below the level of human perception.

## Supporting Information

S1 File RVO model exp data.pdf: Background experimental data regarding the cannulation attempts. (PDF)
