# Supplementary material for: Robotic Assisted Cannulation of Occluded Retinal Veins
Source: PLoS One. 2016 Sep 27;11(9):e0162037. doi: 10.1371/journal.pone.0162037 (PMC5046264; doi:10.1371/journal.pone.0162037)
Supplement: S1 File — (PDF) [file pone.0162037.s001.pdf]

PRECEYES
Medical Robotics

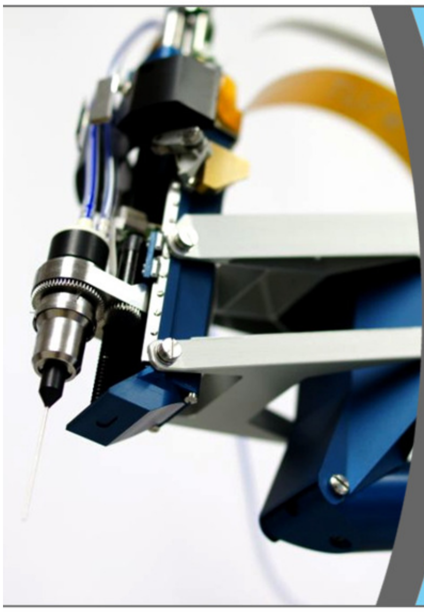

ThromboGenics & PRECEYES
In-vivo retinal vein cannulation experiments

Report of research project 14Q1

May 2014, Marc de Smet, Bart Jonckx, Tom Janssens,
Maarten Beelen, Thijs Meenink, Gerrit Naus

Retinal vein occlusion treatment development

This report presents the results of the research agreement '*Ocriplasmin: retinal vein occlusion model development in the pig and initial feasibility study of enzymatic occlusion dissolving characteristics of Ocriplasmin via vascular delivery*' as agreed upon between Medical Robotic Technologies B.V. and Thrombogenics N.V., d.d. January 1, 2014. In this project, the PRECEYES team has supported Thrombogenics' in-vivo experiments evaluating the efficacy of Microplasmin and has demonstrated the feasibility of reproducible retinal vein cannulation on an in-vivo pig model using micrometer-precision robotic assistance and dedicated glass pipette instrumentation.

In-vivo experiments on January 10, January 31, February 6/7, March 6/10 and April 24/25 have been supported at the Medanex Clinic service facility in Diest, Belgium. During the experiments, the PRECEYES prototype system and corresponding disposable instrumentation, including needles, tubing, trocars, trocar connections, draping, etc. were provided. Furthermore, an E2 laser and endoscopy surgical system from Endo Optiks and an Eye Lite 532 Alcon laser were provided. During the experiments, fulltime technical support by two of our technical experts was available.

Over the course of the experiments, the needle for intravenous treatment of retinal vein occlusions has been improved, optimizing reproducibility of the venous penetration and the subsequent injection. Furthermore, the performance of the PRECEYES prototype system has been tuned, targeting usability in routine operation, increased precision and various hardware and software optimizations for blood vessel penetration. This has resulted in a successfull experimental trial on April 24/25, demonstrating proof of concept of the developed method in combination with Microplasmin to dissolve a retinal vein occlusion.

In the following table, all successful cannulation results, i.e., penetration of a retinal vein, injection of a solvent and clearly visible flow of the solvent in the vein, are summarized. The January experiments were used to train Tom Janssens to perform the cannulation procedure. In most of these experiments, no occlusions were made. The resulting cannulation method is successful in approximately 50% of the attempts, i.e., in 50% of the attempts, a flow of the solvent in the vein is clearly visible. In the other cases, only a minor flow is visible or no flow at all due to erroneous penetration (e.g., penetration next to the vein or subretinal penetration) or squashing of the vein (which is especially relevant in case the penetration attempt is not close to an occlusion). Eventually, a method has been established that allowed for successful cannulation in every specimen (April). The main challenge to a higher success rate most probably is a lack of feedback of successful penetration of the vein, i.e., in these experiments penetration success is only visually inspected by the surgeon.

During the February and the March experiments, different parameters were varied.

1. A reproducible occlusion model was established: developed by Tom Janssens, eventually switching from the use of an extra-ocular laser to an intra-ocular laser in April.
2. A reproducible venous penetration method was developed: this has resulted in a method with a virtual software bound that allows penetrating the vein in a controlled way.
3. Different injection needles have been evaluated: a disposable prototype glass pipette with a metal shielding has been developed that allows for quick changing and robust introduction in the eye.

4. The effect of different injection locations was evaluated: these experiments indicated the need for cannulation directly next to the occlusion, as opposed to in a branch of the occluded vein, ensuring direct contact between the occlusion and Microplasmin.

Appendices

A. Pictures illustrating the cannulation results
B. Notes on the in-vivo experiments
    1. January 10
    2. January 31
    3. February 6 & 7
    4. Experimental plan for March 7 & 10
    5. March 7 & 10

C. Logbook of the in-vivo experiments
    1. February 6 & 7
    2. March 7 & 10
    3. April 24 & 25

D. Phase 1 slides

Appendix A:

Pictures illustrating the cannulation results

Appendix B:

Notes on in-vivo experiments

Appendix B-1:

January 10, notes on in-vivo experiments

Purpose

The goal is to train Tom and Thijs to prepare and perform in-vivo cannulation experiments on a pig model.

New technical features

- Metal trocar holder is aligned for usage of the shielded BRVO needle
- Shielded interchangeable BRVO needle with light fibers, illuminating the needle tip

Setup

- Pigs with non-occluded eyes were used
- Custom (v2) Research Instruments pipettes were used

Results

- Tom was able to prepare a surgical setting
- All vein-penetration attempts failed

Conclusions and remarks

Tom is trained in preparing the pig eye for in-vivo (cannulation) experiments
Thijs is not trained due to lack of time and the urgency for Thijs to support in mechanical issues

- All attempts to penetrate the vein failed:
  - Non-occluded veins are difficult to penetrate, because of
  - Low vein backpressure due to the lack of an occlusion holding the blood inside
  - Only RI pipettes were used, which may possibly be too blunt. => this requires a comparison with other instruments
- Non-collocated mode of the system was tried, i.e., manipulating the motion controller with the left hand to control the instrument manipulator on the right side (during non-collocated mode the Φ and Ψ movements were inverted using the software); seems feasible and relatively easy
- The light strength of the illuminated pipette tip was too low and only seen when the ambient light was turned off (chandeliers and light probe).
  - Use a different light source with higher strength
  - More fibers to transfer the light into the pipette
  - Alternate the bevel angle, most light is reflected back or refracted to emit sideways (radially)
- Changing pipettes must be performed frequently and quickly (keep using a sharp instrument)

Appendix B-2:

January 31, notes on in-vivo experiments

Purpose

The goal is to train Tom and Thijs to prepare and perform in-vivo cannulation experiments on a live pig model.

New technical features

- Angled tip pipette
- Improved instrument

Setup

- Two pigs were used
  - One with occluded eyes was used
  - One non-occluded eye of the second pig was used (the fourth eye wasn't used)

- Pipettes:
  - Custom Research Instruments (RI) pipettes (20µm, 40° bevel tip, titanium metalized)
  - Clunbury Sc. B100-58-30 (with 30 µm, and 30° bevel tip)
  - One B100-58-30 with a 30° angled tip of 0.4 mm

Results

- Tom is trained in preparing the pig eye for in-vivo (cannulation) experiments
- Thijs had a minor training but was able to prepare the eye once
- Multiple vein penetrations, only minor infusion (1 or 2)

Conclusions and remarks

- Tom and Thijs are now able to prepare the eye and to perform RVO cannulation surgery
- The Clunbury Sc. pipettes were able to penetrate the vein
- The RI pipettes need further examination

Future work

- Improve the instrument with trocar and trocar holder guidance, such it won't disrupt the Z-movement

Appendix B-3:

February 6 & 7, notes on in-vivo experiments

Purpose

Reproducible RVO cannulation procedure: method, model and instrumentation.

New technical features

- Improved instrument: straightened
- Improved instrument guidance
- Improved trocars: looser fit

Setup

- 4 pigs were used, with 6 occluded eyes
    - (4x OD, 2x OS)
    - Occlusions in size of 1100-2500 µm
    - Location of occlusion 1500 to 3000 µm from optic nerve
-
- Pipettes:
    - Clunbury Sc. B100-58-30 (

Results

- Total attempts: 23, penetration 11, 2 occlusions solved
    - 6 penetration attempts were made just next to or just on the occlusion. 4 resulted in successful penetrations
- One penetration at a vein over a detachment
- 3 attempts went through and through (TT)
    - One manual TT
    - One penetration of 10 clicks (x50µm)

Summary: In-Vivo experiments					
Attempt	Pig eye	Penetra-tion	Occlusion solved	Instrument	Remarks
06-02-2014					
1	TGpig50 OD	TT		CS-Thin 1	
2		yes	no		Not clear if intravenous injection was made
3		No			
4		No			
5		No			Subretinal, retinal detachment
6		Yes	No		Penetrated vein over retinal detachment
7		No			
8		TT			
9		No		CS-Thin 2	
10	TGpig50 OS	Yes	No	CS-Thick 1	
11		Yes	No		**Penetration near occlusion. (Needle tip pointing perpendicular to vein surface)**
12	TGpig51 OS	Yes	Yes	CS-Thick 1	**Penetration at edge of occlusion. Occlusion solved**
13	TGpig51 OD	Yes	No	CS-Thick 2	

14		No			
15		Yes	No		
07-02-2014					
16	TGpig52 OS	No		CS-Thick 3	
17		Yes	yes	CS-Thick 3	Occlusion solved, **penetration near occlusion**
18	TGpig52 OD	TT	no	CS-Thick 4	TT: When retracted, the needle tip enters the vein and directly filling the vein with µPL
19	TGpig53 OD	No		CS-Thick 5	
20		Yes	No		@ t=13 min (in video), intravenous injection of µPL
21		Yes?	No	CS-Thin 3	
22		Yes	No		
23		Yes	Yes		Manual penetration (@ t = 28 min)**, near the occlusion**. "klonter" is flushed forward". Further, occlusion was already 15 min in contact with µPL (pumprate 80)
Total: 23	6 eyes	12 pentr	3 solved		

Conclusions and remarks

- (again) Positioning is not the problem. Difficult to verify penetration.
  - A light source close by, provides better perception of penetration than global light. This, due to the sharper instrument shadow. The limitation has to do also with:
    - The magnification power that can be achieved with the current microscope, probably as good as we can get
    - Use of the current endoscope did not improve our ability to follow the penetration process or predict when we would enter the retina. For this a different type of endoscope is needed, probably a GRIN lens endoscopic system (in development)
    - Fixation of the light source is a huge benefit In this way, the only movement in the eye at the critical moment is the catheter (pipette). However, the light source should provide a good enough shadow to clearly see it approaching and touching the vessel surface. Even then the magnification appears to weak if in an anterior-posterior direction.

- From the surgeon's perspective, the veins are not too small; the diameter of the catheter (pipette) appears adequate to allow penetration. In most cases, the robot performed well with smooth positioning above the vein, but on occasion the movement could be improved. Z advance was sometimes difficult to control. One option is to consider using the pedal as an actuator for the advance (as a gas pedal).
- Pump was on before injection to push away the back end of the vein during the penetration movement to prevent the instrument going through and through.
  - This did not always work, but did help to know when we entered the vein.

  - We also had situations where we indented the vein, we saw the vein wall spring up around the catheter (pipette) and to our surprise we had a double perforation. In the first case, the pump was not on, in the second it was.

- After going through and through, when the instrument is retracted and the tip comes inside the venous cavity, the vein quickly fills
  - Lower pump rates should be sufficient to fill the vein quickly (remark attempt 18)
  - In contrast, higher pump rates will flush the occlusion forward (attempt 23). A higher pump rate is sometimes also required to see if we are in the vein. With many of the cannulations there appears to be reflux. The reflux can be such that little fluid enters the vein,
- Penetrating the vein just aside or just on the occlusion appears relatively easy:
  - The vein cannot be flattened while penetrating due to the occlusion (This statement needs further validation)
  - 6 attempts near or on the occlusion, 4 successful: 66%
  - 17 attempts on various other locations: 8 successful: 47 %
  - Penetrating in the occlusion itself may be difficult. The walls are damaged, and the clot may or may not accept fluid injection. On one occasion we entered, did not feel that it was successful but upon entering elsewhere, the original perforation site started to bleed. Entering next to the occlusion is best.
- The Clunbury Sc. pipettes performed well in strength. Where in earlier experiments most/all tips broke. Current status:
  - 4 (out of 8) where completely intact; CS-thick 4 and 5, CS-thin 1 and 3
  - 1 only the very tip of the bevel broke; CS-thick 3
  - 2 broke at +/- 50 µm; CS-thick 2, CS-thin 2
  - 1 broke in handling
- No conclusion can be made (yet) regarding sharpness of the various pipettes
- The tip of the instrument should not be positioned right on top of the vein, but with the tip perpendicular to the outer surface of the vein (with respect to the cross-sectional plane of the vein)
  - Subsequently, an angled tip pipette may be use to pierce the vein from the side.
  - At this point, Marc believes this would be the most practical and safe approach. In an XY plane we can see fairly well where we are positioned. Indentation of the vein would be clearly visible and piercing through and through would not put Microplasmin in the subretinal space (when this occurred, the retina turned white which is probably not good).
  - A lateral motion by the robot is not a natural movement that can easily be generated with the motion controller. This might require some degree of automation and programming so that it is triggered by the operator when needed.
- Occlusions made, should to be consistent in size. Results are hard to compare. Moreover, this holds especially if a control group is made. Marc: the ideal size is the one achieved with the last animal, about 1000-1300 µm. These should be small, with only partial occlusion achieved when initially placed. They appear to close completely by the time of the experiment
- Attempt 23 flushed away the occlusion, which might be caused by the enzymatic function of µPL
- **Technical conclusions**:
  - The PRECEYES Surgical System ‘s precision and usage are adequate

- The improved guidance of tool through the trocar worked. The Z-movement was much smoother. But could still be improved.
- X, Y and Z adjustments by hand are a little inconvenient (and time consuming, however that is about a minute)
- Non-collocated mode of the system was used i.e., manipulating the motion controller with the right hand to control the instrument manipulator on the left side. During non-collocated mode the Φ and Ψ movements were inverted using the software
- Changing pipettes must be performed frequently and quickly (to keep using a sharp instrument)

Pictures of in-vivo experiments, February 6 & 7

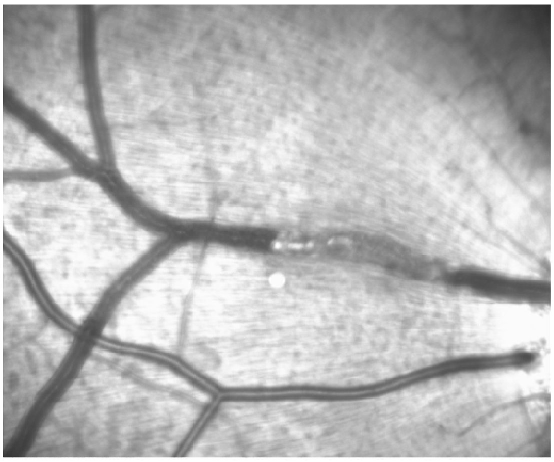

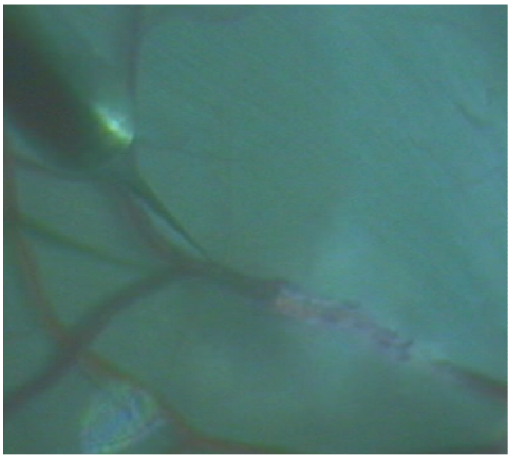

Figure 1: Pig 50, OD right eye. Left, a fluor image of the occlusion with all attempt locations indicated. On the right the microscope image of attempt 1. None of the attempts resulted in a penetration.

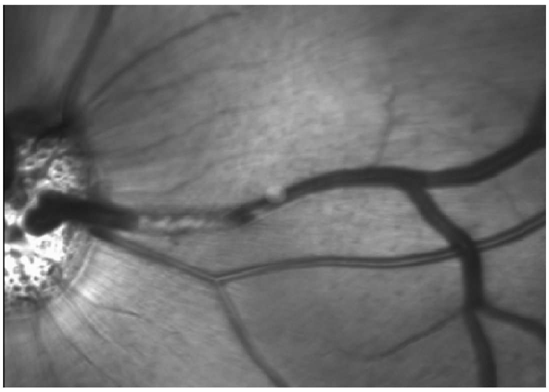

Figure 3: Pig 51, OS left eye. A fluor image of the occlusion with all attempt locations indicated, all penetration attempts were successful.

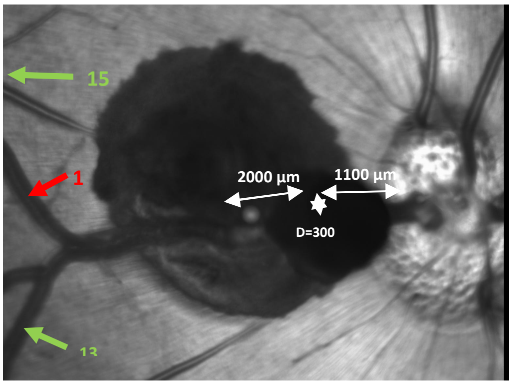

Figure 3: Pig 51, OD Right eye. A fluor image of the occlusion with all attempt locations indicated, attempt 13 and 15 showed successful penetration.

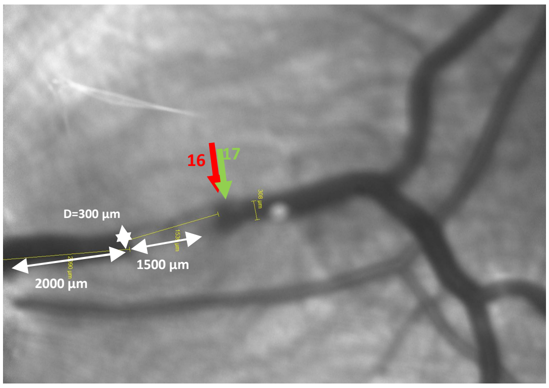

Figure 5: Pig 52, OS Left eye. A fluor image of the occlusion with all attempt locations indicated. Attempt 17 showed an successful penetration, that resolved the occlusion.

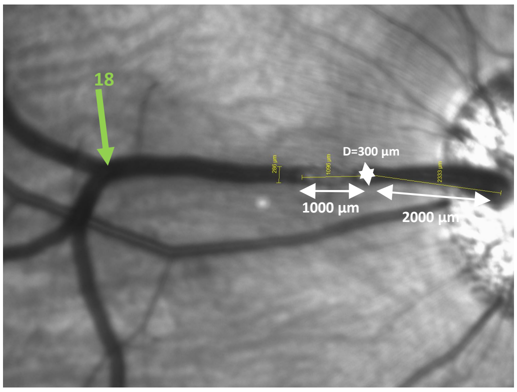

Figure 5: Pig 52, OS Left eye. A fluor image of the occlusion with all attempt locations indicated. Attempt 18 went through and through. However, on retract, the tip was intravenous and injection µPL.

Appendix B-4:

Experimental plan for March 7 & 10

Experimental plan
From cannulation experiments performed 6-7 February, we concluded that *penetrating the vein at or near the occlusion was most successful.* The focus of these experiments should be to:

- Further explore and improve the cannulation procedure near the occlusion.
- Reproduce results of last experiments to underpin the conclusion above.

To show reproducibility, variables should be similar to the experiments on 6-7 February, a.o. these are: occlusion size (as the shortest made) and location, instrument pipettes and approach/method.

Planning/protocol

Pig preparation

- Occlusions as short as possible, though the veins must be occluded
    - Measure occlusion size
    - Validate occlusion
- Occlusions made at 1500 µm
    - Measure location occlusion
- Measure vein diameter

    - After occlusion
    - After branching of vein (measure both branches)
    - In front of vein branching
    - measure the distance
- Take (fluo) images for documentation

Penetration/cannulation planning

- Choose possible penetration locations using the fluo images, and number them. For each location:
    - Determine the distance from the ON (for documentation)
    - [illegible]
- Per eye, the first locations that must be validated should be next to the occlusion at the occluded side

Cannulation procedure

- µPL pump off(!)
- Position the tip of the instrument (pipette) at the location as planned
    - The centre line of the instrument should intersect with the centre line of the vein

- Touch the vein (gently) at the planned location
    - Use the visual indentation to verify step 5 and 6
- Piercing the vein:

- µPL pump on
- Visually inspect the penetration has succeeded
- If the attempt fails start the penetration procedure over from step 4. Do not make a subsequent attempt, from the position made after step 9!

Appendix B-5:

March 7 & 10, notes on in-vivo experiments

Purpose

1. Reproducible RVO cannulation procedure: method, model and instrumentation.
2. Make cannulation near the occlusion consistently successful

New technical features

- New penetration motion 'single-shot':
- Further improvement of instrument guidance

Setup

- 4 pigs : 3 eyes on Friday, 4 eyes on Monday
    - Preferred occlusion length: 1000-1500 µm
    - Location of occlusion: start at 1500 µm
-
- Pipettes:
    - Clunbury Sc. B100-58-30 (CS-Thick, with 30 µm tip, and 30° bevel tip, titanium metalized)
    - Clunbury Sc. B100-70-30 (CS-Thin, with 30 µm tip, and 30° bevel tip, titanium metalized)
    - Clunbury Sc. B100-58-30 with the last 0.5 mm angled at 30°

Results

- Total penetration attempts: 29, successful penetration 15, 3 occlusions solved
    - 6 penetration attempts were made just next to or just on the occlusion. 4 resulted in successful penetrations

- One penetration at a vein over a detachment
- 1 attempt went through and through (TT)
    - One manual TT

Summary: In-Vivo experiments					
Attempt	Pig eye	Penetra-tion	Occlusion solved	Instrument	Remarks
07-03-2014					
1	TGpig65 OS	No		CS-Thick 1	
2		TT			Next to occlusion, ablation made
3		No		CS-Thick 2	
4		yes	**Yes**		Next to occlusion. Multiple times vein filled with µPL(low pumprate p20(=13.2µL/min). **Occlusion solved over time!**
5	TgPIG65 OD	yes		CS-Thick 3	On occlusion
6	TGpig65 OD	Yes			
7		yes			Next to occlusion. Judgement of penetration still difficult.
8		yes	**yes**		Slight manual rubbing the vein, after penetration motion. **Occlusion solved (low pumprate p20(=13.2µL/min))**
9		yes			After occlusion
10	TGpig66 OD	Yes	No	CS-Thin 1 old	Between occlusions (two small occlusions)
11		Yes	No		
12		Yes	No		
10-03-2014					

13	TGpig67 OS	No	No	CS-Thin 2 old	
14		No			
15		No			
16		yes	No		
17		No		CS-Thin 3new	Manual attempt
18		No			
19	TGpig67 OD	No		CS-Thin 4new	
20		yes	no		Manual attempt + xy rubbing
21		Yes	**yes**		Manual next to occlusion, Air in the fluidic system pushed the occlusion
22		Yes	**(Yes)**		Subsequent attempt, manual, there is air visible after the occlusion. Pump is a few times put off to refill the veins with blood.
23		Yes	-		Manual rubbing
24	TGpig68 OD	No		Curved pipette 1	A pipette with a curved pipette was used. Manual penetration attempt
25		No			Manual penetration attempt
26		Yes	no		Manual penetration attempt
27	TGpig68 OS	No		Curved pipette 2	Manual penetration attempt
28		No			Manual penetration attempt
29		No			Manual penetration attempt
Total: 29	7 eyes	15 pentr	3 solved		

Conclusions and remarks

The goal was to define a reproducible vein penetration method

- The results of 6-7 February are reproduced: 15 successful penetrations out of 29 attempts, 52%
    a) 4 attempts next to an occlusion, 2 on an occlusion, 5 successful penetrations, 83%.
        i) Although the figures show reproducibility in the method of penetrating the vein next to an occlusion, the subjective perception/opinion of the user do support the numbers conclusively!
    b) 6 (axial) penetration attempts where done manually, 4 successful penetration, 67%.
        i) Here, all penetrations were made because the user performed a (x and y) rubbing motion onto the surface of the vein.
    c) 6 penetration attempts were done manually because of a curved tip, 1 successful penetration, 17%
- The goal to define a reproducible and consistent method to penetrate the vein <u>is not met!</u>
    a) i.e. at this point it cannot be predicted when the vein is/will be penetrated.

In the first two eyes (TGPIG65 OD and OS) the occluded veins were penetrated and subsequently cannulated using a low pump rate: P20 = 13.2 µL/min. Multiple times the pump was disabled to refill the vein with blood and µPL alternately. In time both occlusions appeared to be solved and blood was flowing.

3) Design an instrument with a shoot mechanism

4) Ad a vibrator to the instrument to rub the venous wall at high speed (or ultrasonically actuated), or
5) Program a vibration overlay to the instrument movement.
6) Program a user motion boundary to manually:
    a. Rub the venous wall to penetrate the vein, or
    b. Push the pipette tip along the direction of the vein

Pictures of in-vivo experiments, March 7 & 10

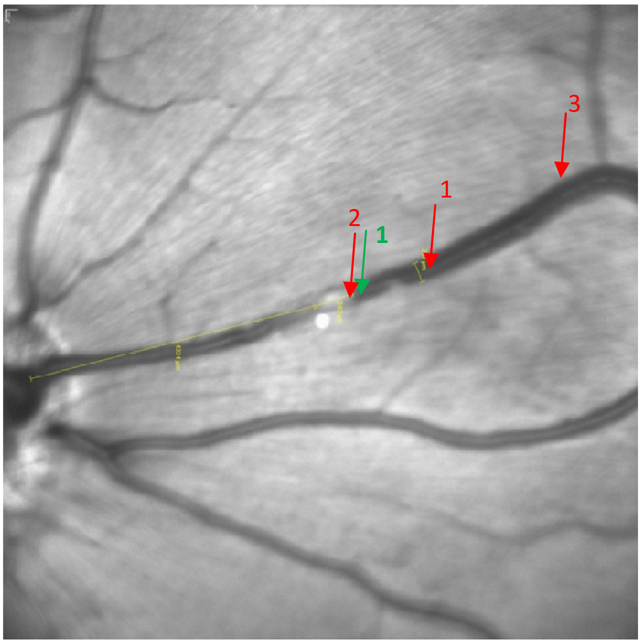

Figure 1: Pig 65, OS. On the left, a fluo image of the occlusion with all attempt locations indicated, attempt 4 successful penetration.

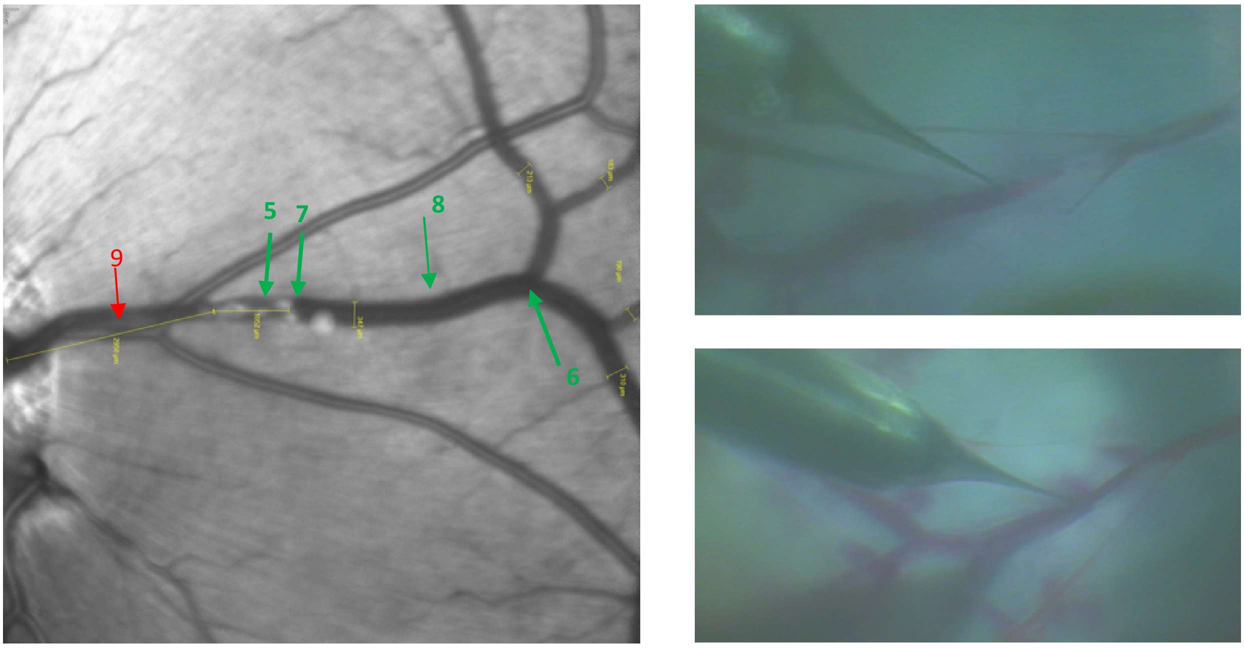

Figure 2: Pig 65, OD. On the left, a fluo image of the occlusion with all attempt locations indicated, attempt 5-8 show successful penetrations. On the right: Top image, vein with occlusion, lower image, same vein with occlusion solved.

Pig 66 OD => no fluo image available, **Pig 66 OS** no occlusion made.

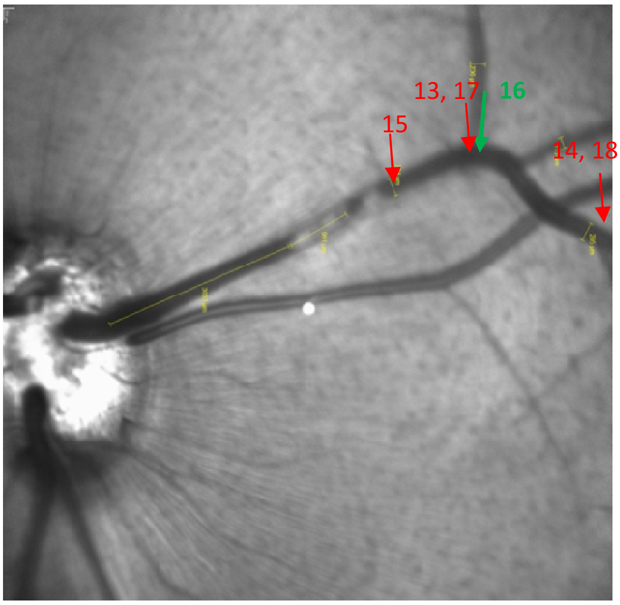

Figure 9: Pig 67, OS. A fluo image of the occlusion with all attempt locations indicated, attempt 16 shows a successful penetration.

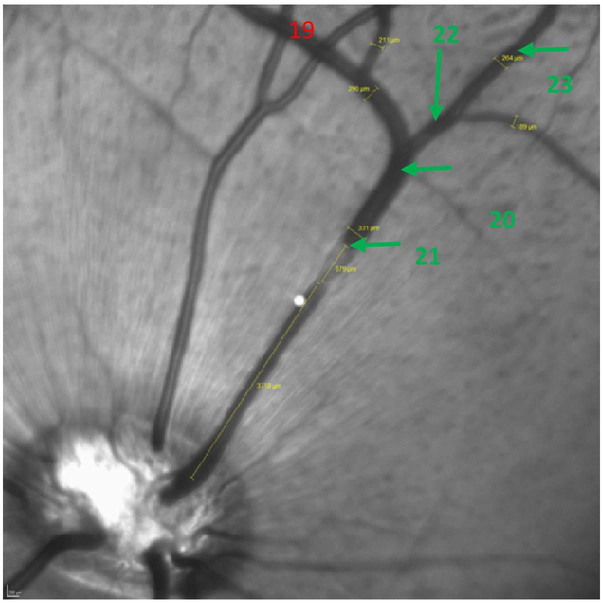

Figure 9 Pig 67, OD. A fluo image of the occlusion with all attempt locations indicated. Attempt 19 is at an vein-artery crossing. Attempt 20-23 show successful penetrations.

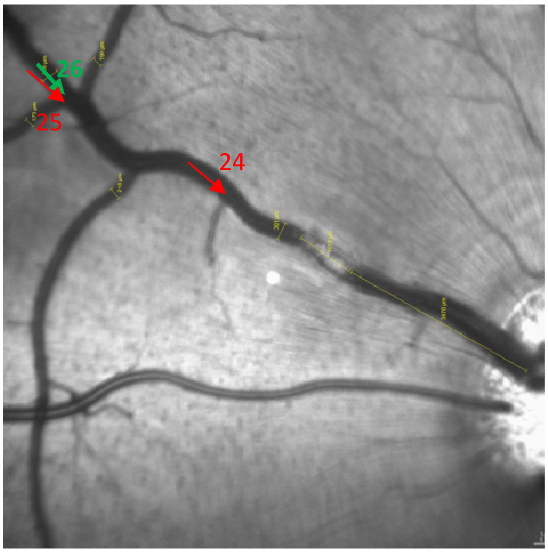

Figure 11 Pig 68, OD. A fluo image of the occlusion with all attempt locations indicated. A pipette with an angled tip was used. Attempt 26 shows a successful penetration.

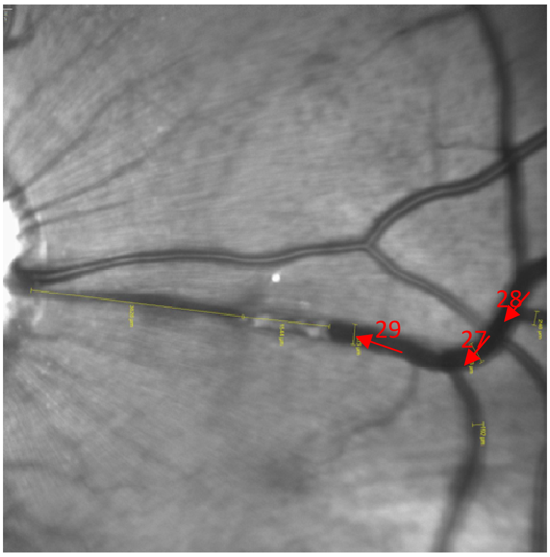

Figure 11 Pig 68, OS. A fluo image of the occlusion with all attempt locations indicated. A pipette with an angled tip was used. No penetration was made.

Appendix C:

Logbook of in-vivo experiments

1. *February 6 & 7*
2. *March 7 & 10*
3. *April 24 & 25*

**February 6, 2014**	Attempt 1	Attempt 2	Attempt 3	Attempt 4
Eye #, right / left	Pig50, OD (right eye)	Pig50, OD	Pig50, OD	Pig50, OD
User	Mds	MdS	MdS	MdS
Start time / date	6-feb-2014 11:00	@ 11 min	@25 min	@30min
Video from microscope<br>Filename / time in movie	…11-10-12.mpg			
Tool (RI, CS -thin, CS -thick)	CS -Thin, 1	CS -Thin, 1	CS -Thin, 1	CS -Thin, 1
Occlusion size	L=2800 µm, d=300µm			
Occlusion location in eye	1900 µm from NO (nervus opticus)			
Vein size (diameter)	300 µm,	+/- 200 µm	300 µm	+/- 150 µm
Penetration location, relative to occlusion	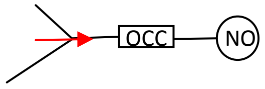	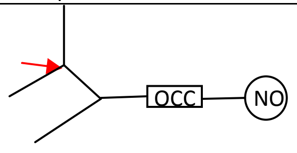 @ 2nd Y-split	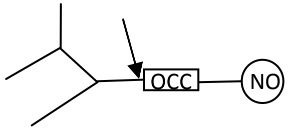 Next to occlusion	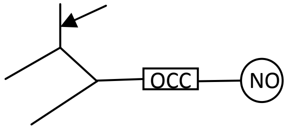
Approaching angle	+/- 80 deg	"	"	"
Tool bevel orientation	Not Noticable	NN	NN	NN
Number of piercing movements (x50µm)	50µm, manual	2	2	3
Penetration<br>(Succeeded / subretinal)	Subretinal, hemmorage	yes		
Injection:(Intravitreal / flow visible in vein)	Intravitreal	Intravenous(?)	Intravitreal	Intravitreal
Injection rate	P30	P30		P60
Total volume injected (µL)				
Occlusion resolved?				
Hemmorrhage?	Yes	Yes		
Remarks<br>(if attempt cancelled, explain why)	Through and through (TT) the vein<br>Vitrectomy 11:15<br>Easy penetration (manual performed.	In vein => bleeding<br>After vitrectomy<br>(possibly vein filled with µPL (microplasmine), vein from OCC to NO is blank.)	(use of contact lens)<br>Light bleeding	Not in vein, against vein
Conclusion	Positioning is not the problem, the veins are that small it is difficult to notice penetration.<br>Attempt 3: Pierce just next to occlusion, the occlusion holds the shape of the vein, not flattening it while penetrating (though failed).			

**February 6, 2014**	Attempt 5	Attempt 6	Attempt 7	Attempt 8
Eye #, right / left	Pig50, OD (right eye)	Pig50, OD	Pig50, OD	Pig50, OD
User	Mds	MdS	TJ	TJ
Start time / date	@33	@35	@4 min	@8.30min
Video from microscope Filename / time in movie	2014_2_6_11-10-12.mpg		2014_2_6_11_57_30.mpg	
Tool (RI, CS -thin, CS -thick)	CS-Thin, 1	CS -Thin, 1	CS -Thin, 1	CS -Thin, 1
				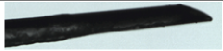 intact
Occlusion size	L=2800 µm, d=300µm			
Occlusion location in eye	1900 µm from NO (nervus opticus)			
Vein size (diameter)	+/- 200 µm	+/- 100 µm	250 µm	250 µm
Penetration location, relative to occlusion	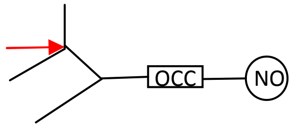 @ 2nd Y-split	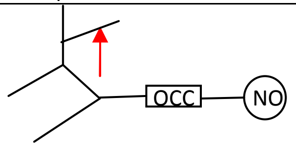	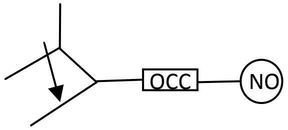	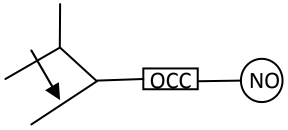
Approaching angle	+/- 80 deg	"	"	"
Tool bevel orientation	Not Noticable	NN	NN	NN
Number of piercing movements (x50µm)	50µm, 2	4	5 of 25 µm, 7	10 of 50 µm
Penetration (Succeeded / subretinal)	No	Yes		TT
Injection:(Intravitreal / flow visible in vein)	Subretinal	Flow of µPL away from the OCC (not through)		
Injection rate	P30	P50-70	P40	P60
Total volume injected (µL)				
Occlusion resolved?				
Hemmorrhage?	Yes	Yes, at retract		
Remarks (if attempt cancelled, explain why)	Retinal detachment<br>A lot of air (from the instrument)	Penetration while injecting µPL<br>Penetration attempt at vein over ablation.<br>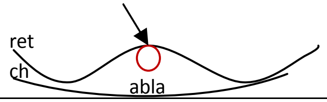	Blunt pipette? => *no! see picture attempt 8*	TT => problem, flat pushed vein, through both walls<br>While retracting the instrument the tip became in the vein and directly
Conclusion	Inject while penetrating => @ penetration, flow is directly visible, because veins become blank. It may also prevent TT, by pushing the tissue forward of the extrusion (healon effect)			

**February 6, 2014**	Attempt 9	Attempt 10	Attempt 11	Attempt 12
Eye #, right / left	Pig50, OD (right eye)	Pig51, OS (left eye)	Pig51, OS (left eye)	Pig51, OS (left eye)
User	TJ	MdS	MdS	MdS
Start time / date	@ 24	6-feb-2014 16:15 @0 min	@3 min	@17 min
Video from microscope<br>Filename / time in movie	2014_2_6_11_57_30.mpg	2014_2_6_16_15_44.mpg		
Tool (RI, CS-thin, CS-thick)	CS-Thin, 2	CS-Thick, 1	CS-Thick, 1	CS-thick, 1
	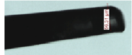 broke @ 55 µm			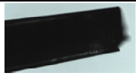 Broke in handling
Occlusion size	L=2800 µm, d=300µm	L=1100 µm d=300 µm		
Occlusion location in eye	1900 µm from NO (nervus opticus)	@ 1000 µm from NO,		
µVein size (diameter)	+/- 200 µm	+/- 200 µm	250 µm	
Penetration location, relative to occlusion	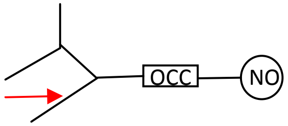	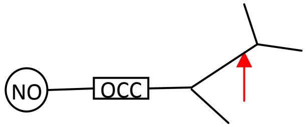<br>before 2nd Y-split	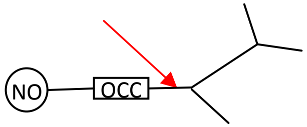	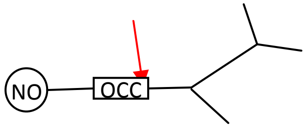<br>@ start of occlusion
Approaching angle	+/- 80 deg	"	+/- 45 deg 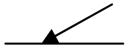	
Tool bevel orientation	Not Noticable	NN	NN	
Number of piercing movements (x50µm)	50µm, 10 and 8 times	4	5 of 25 µm, 7	1
Penetration<br>(Succeeded / subretinal)		yes	yes	yes
Injection:(Intravitreal / flow visible in vein)		?	flow visible	Flow visible in opposite direction
Injection rate	P60	P50	P40, P80 @ 10 min	P60
Occlusion resolved?			no	no
Hemmorrhage?	Y	Small bleeding		
Remarks<br>(if attempt cancelled, explain why)	New pipette<br>Same location<br>Pomp while penetrating (PWP)		@ 9 min pomp on again<br>Approach more from the side of the vein (image below)<br>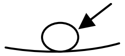<br>@12:30 out	
Conclusion	Possible strategy: Pierce at (the edge) of the occlusion, fill the veins and wait for the enzymatic working of µPL (as attempt 12). Note: (attempt 11) when approaching the vein somewhat radially touch the vein as such, i.e. not at the top of the vein, but perpendicular to the vein.<br>Possible solution: approach the vein from the side using a angled tip, the vein is pushed sideways not flattened, preventing a TT.			

February 6, 2014	Attempt 13	Attempt 14	Attempt 15	Attempt
Eye #, right / left	Pig51 OD (right eye)			
User	MdS	MdS	MdS	
Start time / date	6-feb-2014 17:45 @4	@12		
Video from microscope Filename / time in movie	2014_2_6_17_45_16.mpg			
Tool (RI, CS-thin, CS-thick)	CS-thick, 2	CS-thick, 2	CS-Thick 2	
			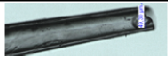 broke @ 50µm	
Occlusion size	L=2000µm,d=300µm (large trauma)			
Occlusion location in eye	1500µm			
Vein size (diameter)				
Penetration location, relative to occlusion	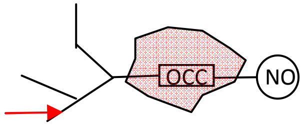	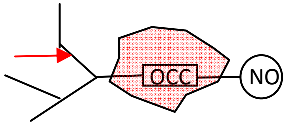	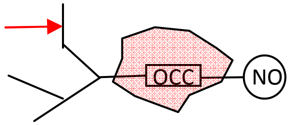	
Approaching angle	+/- 80 deg			
Tool bevel orientation				
Number of piercing movements (x50µm)	2	4	5	
Penetration (Succeeded / subretinal)	Yes @ 5 min	no	Yes	
Injection:(Intravitreal / flow visible in vein)	Flow in opposite direction		Slow filling of vein	
Injection rate	P40	P40	P40	
Total volume injected (µL)				
Occlusion resolved? (n/a, yes, no, injection time)	no			
Remarks (if attempt cancelled, explain why)	Use of endoscope for additional feedback. => the light close by gave better dept perception, shadow was more pronounced present	endo	endo	
Conclusion	The use of a light source close by (@ a few mm) will give better perception of penetration, due to sharper instrument shade.			

**February 7, 2014**	Attempt 16	Attempt 17	Attempt 18	Attempt 19
Eye #, right / left	Pig52 (091932) OS (Left eye)		Pig52 (091932) OD (Right eye)	Pig53 OD (Right eye)
User	MdS	MdS	MdS	MdS
Start time / date	7-feb-2014 10:52 @4	?	@ 7 min micro, @ 4 min endo	14:00
Video from microscope<br>Filename / time in movie	Endo: Video_20140207_105245.wmv		2014_2_7_12_18_14.mpg<br>End: 20140207_122002.wmv	
Tool (RI, CS-thin, CS-thick)	CS-thick, 3	CS-thick, 3	CS-thick, 4	CS-thick, 5
Tool quality after surgery		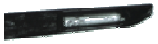 very tip broke	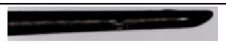 intact	
Occlusion size	L=1500µm,d=300µm		L=1000µm,d=300µm	4 to 5 times vein diameter, (300 µm) =>1500µm
Occlusion location in eye	2000µm from NO		2000µm from NO	3000µm from NO
Vein diameter @ penetration				200µm
Penetration location, relative to occlusion	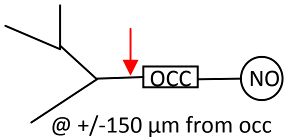<br>@ +/-150 µm from occ	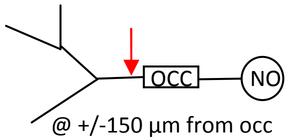<br>@ +/-150 µm from occ	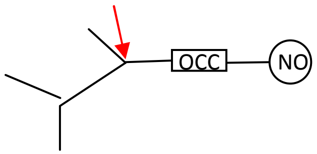	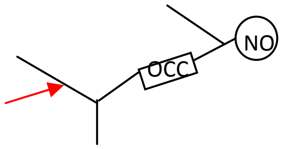
Number of piercing movements (x50µm)	2	2	2,	3
Penetration (Succeeded / subretinal)	No, just above	Yes	TT, subretinal	No
Injection:(Intravitreal / flow visible in vein)		Flow visible	TT, at retract intravenous, flow @7 min	
Injection rate	P40	P40	P40, a@ 4 min P60	P40
Total volume injected (µL)		Tot 200 µL	Tot 150 µL	
Occlusion resolved?<br>(n/a, yes, no, injection time)	no	Yes	No	
Hemmorrhage?	Yes	a little		
Remarks<br>(if attempt cancelled, explain why)	+ endo, +chandelier<br>First inject µPL to induce a vitrous detachement +<br>-use of endo for light (sharp shadow and local vision	After a while µPL visible in vein up to NO<br>-after 2 penetrating clicks, x-y motion (and possible Z) => direct flow!! Like "cutting" through vein	- Through and Through (TT), at retract the tip enters the vein cavity, subsequently the vein fills quickly with µPL<br>-small hole in retina	Vitrectomy<br>(no fluor image available
Conclusion	Sometimes the penetration goes TT, subsequently when retracting the instrument (while injecting) the tip of the instrument comes in the venous cavity, quickly filling the vein with µPL (within second). => this may conclude the pump rate is too high.			

**February 7, 2014**	Attempt 20	Attempt 21	Attempt 22	Attempt 23
Eye #, right / left	Pig53 OD (Right eye)			
User	MdS	MdS	MdS	MdS
Start time /date /time in movie	7-feb-14, 14:00 @13 min	@ 20		@28 min
Video name:<br>Fluor image name:				
Tool (RI, CS-thin, CS-thick)	CS-thick, 5	CS-thin, 3	CS-thin, 3	CS thin 3
Tool quality after surgery	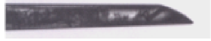 intact			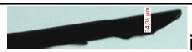 intact
Occlusion size	4 to 5 times vein diameter, (300 µm) =>1500µm			
Occlusion location in eye	3000µm from NO			
Vein diameter @ penetration	200µm	200 µm	100 µm	300µm
Penetration location, relative to occlusion	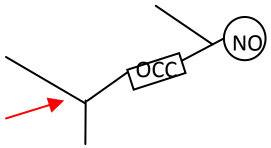	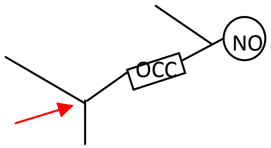	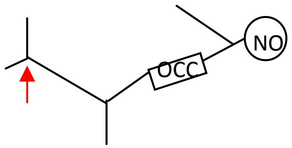	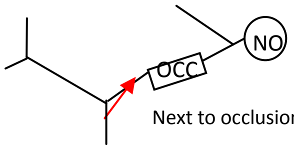 Next to occlusion
Approaching angle				
Tool bevel orientation				
Number of piercing movements (x50µm)	1	2	Manual	Manual
Penetration<br>(Succeeded / subretinal)	yes	maybe	Yes	Yes
Injection:(Intravitreal / flow visible in vein)	Yes, vein filled with µPL		Intravenous	intravenous
Injection rate	P40	P60	P40	P80
Total volume injected (µL)				+/- 300 µL
Occlusion resolved?<br>(n/a, yes, no, injection time)				Yes
Remarks<br>(if attempt cancelled, explain why)	Left and right filled (?)<br>Tip broke<br>(instrument change @ 15 min)	- New instrument<br>- possible penetration @ 22 min		Occlusion “klonter”is pushed forward towards the NO. Occlusion already 15 min in contact with µPL
Conclusion				

**March 7, 2014**	Location: Attempt: 1	Location: Attempt:2	Location: Attempt:3	Location: Attempt: 4
Eye #, right / left	TGpig65 OS			
User	TJ			
Start time /date /time in movie	10:30 @4 min	10:45		
Video name:<br>Fluor image name:	2014-3-7-11-37-42.mpg			Oa. 2014-31-7-11-37-42
Tool (RI, CS-thin, CS-thick)	CS-Thick 1		CS-Thick 2	
Tool quality after surgery	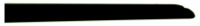 a fracture on the cutting edge		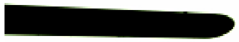 intact	
Occlusion size	580µm			
Occlusion location in eye	4300µm from NO			
Vein diameter @ penetration	300µm			
Penetration location, relative to occlusion	@ 600 µm from OCC 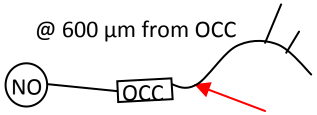	@ 200 µm from OCC 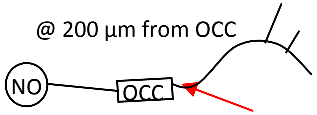	@ ablatio 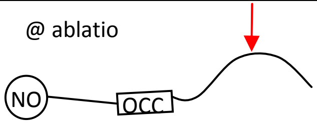	@ 400µm 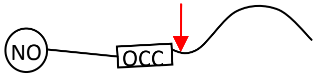
Approaching angle				
Tool bevel orientation				
Number of piercing movements (µm)	2x 150 µm	2x 200µm + 4x 50µm		1x200µm
Penetration<br>(Succeeded / subretinal)	No	TT	no	yes
Injection:(Intravitreal / flow visible in vein)	No	Subretinal => ablatio		Flow visible
Injection rate				P20
Total volume injected (µL)				Vtot 200µL
Occlusion resolved?<br>(n/a, yes, no, injection time)				Yes at 11:40 visible
Hemmorrhage?				
Remarks<br>(if attempt cancelled, explain why)	Pump start at 0.8 m:	-air in vein	Penetration on ablatio	In vein at 11:20 (time) p20<br>P10 at 11:23<br>Poff at 11:25 => P20 at 11.28<br>Poff 11:30 waiting on flow<br>=>repeated a 3x<br>At 11:40 occlusion open P80
Conclusion				

**March 7, 2014**	Location: Attempt: 5	Location: Attempt:6	Location: Attempt:7	Location: Attempt: 8
Eye #, right / left	TGpig65 OD			
User	TM	TM	TM	TJ
Start time /date /time in movie	13:3	@8min40	@14min	@37min
Video name:<br>Fluor image name:	2014-3-7-13-3-58.mpg			
Tool (RI, CS-thin, CS-thick)	CS-Thick 3			
Tool quality after surgery	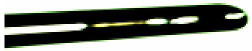 intact			
Occlusion size	580µm			
Occlusion location in eye	00µm from NO			
Vein diameter @ penetration	300µm			
Penetration location, relative to occlusion	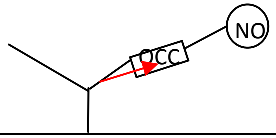	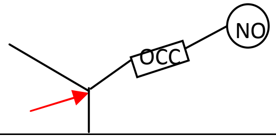	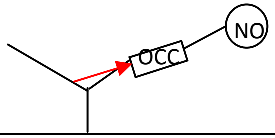	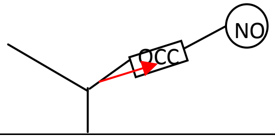
Approaching angle				
Tool bevel orientation				
Number of piercing movements (µm)	1x150µm	1	1	1
Penetration<br>(Succeeded / subretinal)	yes	yes	Yes	yes
Injection:(Intravitreal / flow visible in vein)		Light flow visible@8min vid.		@37.30 flow
Injection rate	P40			P40 p20
Total volume injected (µL)			0.2 mL tot	
Occlusion resolved?<br>(n/a, yes, no, injection time)				Yes, occlusion looks to be moving @26min, normal flow visible @34 min. @ 38.22 flow in both dir.
Hemmorrhage?			Slight hemmorrhage	
Remarks<br>(if attempt cancelled, explain why)	Penetration on occlusion<br>Penetration not noticeable because flow is not visible	Vitrectomy for air bubles		Slight x-y movement to get in vein<br>In vein at 37 min, out of vein at 40 min . refilled with blood at 41 min
Conclusion	It appears that the vein is easier to penetrate when the tip is manually moved(x-y), rubbing the venous wall, while applying pressure on the vein.			

**March 7, 2014**	Location: Attempt: 9	Location: Attempt:10	Location: Attempt:11	Location: Attempt: 12
Eye #, right / left	TGpig65 OD	TGpig66 OD		
User	TJ	TJ	TJ	TJ
Start time /date /time in movie	@45min			@37min
Video name:<br>Fluor image name:	2014-3-7-13-3-58.mpg	Geen	2014-3-7-15-42-9	
Tool (RI, CS-thin, CS-thick)	CS-Thick 3	CS-thin 1 oud		
Tool quality after surgery		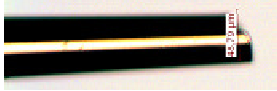 broke at 50 µm		
Occlusion size	580µm	2x 300µm		
Occlusion location in eye	00µm from NO	@4000µm		
Vein diameter @ penetration	300µm	300µm		
Penetration location, relative to occlusion	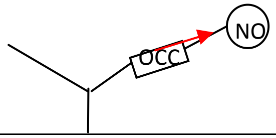	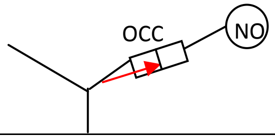	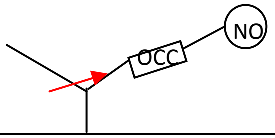	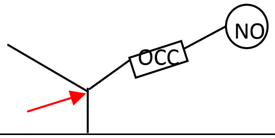
Approaching angle				
Number of piercing movements (µm)	1x150µm +manually advancing	2x150µm	1 + manually rubbing the vein	1
Penetration<br>(Succeeded / subretinal)	yes	Yes	Yes	yes@8:50
Injection:(Intravitreal / flow visible in vein)			Yes, starting directly in video	@9minflow
Injection rate	P40		P20	p20
Total volume injected (µL)				Vtot 0.1 mL (pig 66 OD)
Occlusion resolved?<br>(n/a, yes, no, injection time)				Yes (difficult to judge from vid)
Hemmorrhage?		Slight bleeding		
Remarks<br>(if attempt cancelled, explain why)	Penetration after the occlusion<br>The occlusion looks advanced somewhat.<br>Pentration method: pierce than advance tip along the vein, push the venous tissue up/forward and penetrate.	Penetration between occlusions slight bleeding at 2nd attempt	Penetration at 15u40<br>@1:20 Ablation+subretinal hemorrhage	First air Injection followed by µPL<br>@9.50 Poff => refill of blood<br>@1037 P20 (blood flows out of earlier penetrated hole<br>@11min50 Poff
Conclusion				

**March 10, 2014**	Location: Attempt:	Location: Attempt:	Location: Attempt:13	Location: Attempt: 14
Eye #, right / left	TGpig66 OS		TGpig67 OS	
User	TJ		MS	
Start time /date /time in movie	9u15 10-3-2014		12.50	
Video name:<br>Fluor image name:			2014-3-10-12-53-21	
Tool (RI, CS-thin, CS-thick)	CS-thin 2 old			
Tool quality after surgery	Broke while handling afterwards			
Occlusion size	No occlusion			
Occlusion location in eye				
Vein diameter @ penetration				
Penetration location, relative to occlusion			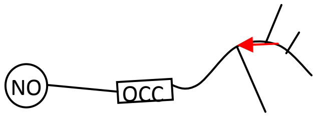	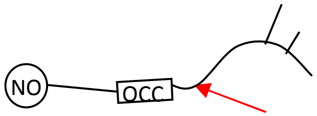
Approaching angle				
Tool bevel orientation				
Number of piercing movements (μm)				
Penetration<br>(Succeeded / subretinal)				150 + 3x50μm
Injection:(Intravitreal / flow visible in vein)				
Injection rate				
Total volume injected (μL)				
Occlusion resolved?<br>(n/a, yes, no, injection time)				
Hemmorrhage?				
Remarks<br>(if attempt cancelled, explain why)			Pipette occluded(?)	Pipette occluded(?)
Conclusion				

**March 10, 2014**	Location: Attempt: 15	Location: Attempt:16	Location: Attempt:17	Location: Attempt: 18
Eye #, right / left	TGpig67 OS		TGpig67	
User	MdS		MS	
Start time /date /time in movie	9u15 10-3-2014		12.50	
Video name:<br>Fluor image name:				2014-3-10-12-53-21
Tool (RI, CS-thin, CS-thick)	CS-thin 2 old		CS-thin 3 new	
Tool quality after surgery			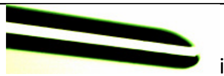 intact	
Occlusion size	1000µm			
Occlusion location in eye	3700µm			
Vein diameter @ penetration	300µm			
Penetration location, relative to occlusion	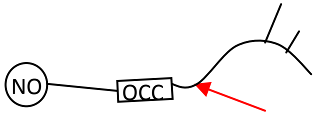	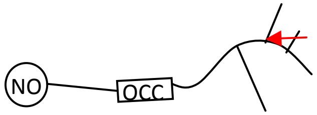	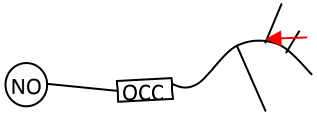	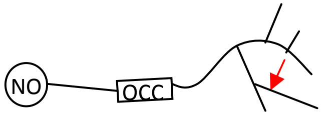
Approaching angle				
Tool bevel orientation				
Number of piercing movements (µm)			Manual	150µm
Penetration<br>(Succeeded / subretinal)		yes		
Injection:(Intravitreal / flow visible in vein)		no		
Injection rate				P50
Total volume injected (µL)				Tot 0.5mL
Occlusion resolved?<br>(n/a, yes, no, injection time)				
Hemmorrhage?			yes	yes
Remarks<br>(if attempt cancelled, explain why)	Pipette occluded	Pipette occluded		
Conclusion				

**March 10, 2014**	Location: Attempt: 19	Location: Attempt:20	Location: Attempt:21	Location: Attempt: 22
Eye #, right / left	TGpig67 OD			
User	MdS			
Start time /date /time in movie	14u32 10-3-2014			
Video name:<br>Fluor image name:	2014-3-10-14-32-38			
Tool (RI, CS-thin, CS-thick)	CS-thin 4 new			
Tool quality after surgery	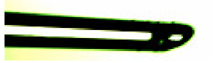 intact			
Occlusion size				
Occlusion location in eye				
Vein diameter @ penetration				
Penetration location, relative to occlusion	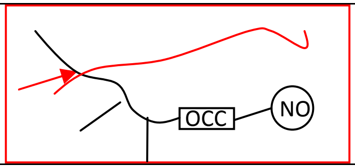	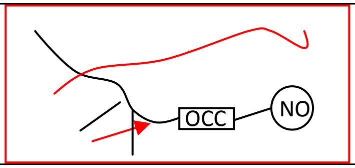	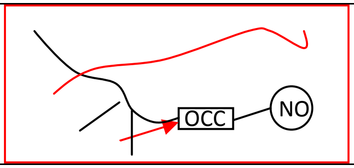	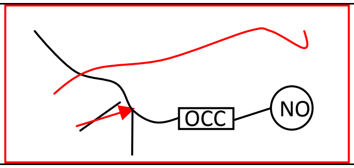
Approaching angle				
Tool bevel orientation				
Number of piercing movements (µm)		Manual+xy	Manual +1x150	
Penetration<br>(Succeeded / subretinal)				
Injection:(Intravitreal / flow visible in vein)		Yes		Flow visible
Injection rate	P50	P80+p40	P80	P80-p20
Total volume injected (µL)				At this attempt 0.1mL
Occlusion resolved?<br>(n/a, yes, no, injection time)			Yes, by air bubles(?)	Yes, air bubles after occlusion
Hemmorrhage?	small	small		
Remarks<br>(if attempt cancelled, explain why)	Penetratie op cruising vein/artery	Pushing the tip along vein<br>@2.30P40<br>@3.50 P80	@19 min air in vein<br>ablatio	@25:50<br>@26:50 P20, @26:55 Poff<br>@27:40 P20, @27.45 Poff<br>Further 3x P20 and Poff, filling the vein with µPL and blood
Conclusion				

**March 10, 2014**	Location: Attempt: 23	Location: Attempt:24	Location: Attempt:25	Location: Attempt: 26
Eye #, right / left	TGpig67 OD	TGpig 68 OD		
User	MdS			
Start time /date /time in movie	14u32 10-3-2014		@20min	@24 min
Video name:<br>Fluor image name:	2014-3-10-15-11-5	2014-3-10-16-17-4		
Tool (RI, CS-thin, CS-thick)	CS-thin 4 new	Curved pipette 1		
Tool quality after surgery		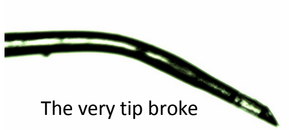		
Occlusion size		1000µm (2e attempt)		
Occlusion location in eye		@3500µm		
Vein diameter @ penetration				
Penetration location, relative to occlusion	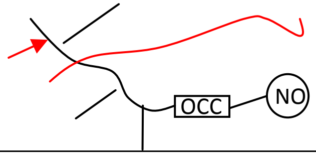	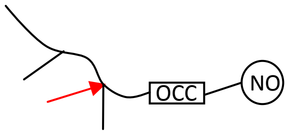	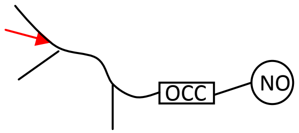	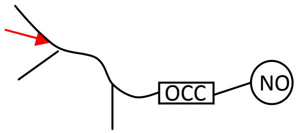
Approaching angle				
Tool bevel orientation				
Number of piercing movements (µm)	150µm + manual rubbing	Manual	Manual	Manual
Penetration<br>(Succeeded / subretinal)	yes			Yes
Injection:(Intravitreal / flow visible in vein)	@ 1min			yes
Injection rate	P50	P40		P40-p20
Total volume injected (µL)		(start syringe 0.52 mL)		0.22mL
Occlusion resolved?<br>(n/a, yes, no, injection time)				Yes, air bubles after occlusion
Hemmorrhage?		small		
Remarks<br>(if attempt cancelled, explain why)	Penetrating 150µm then manually advancing/penetrating and rubbing the vein	Pushing the tip along vein<br>@2.30P40<br>@3.50 P80		@26:30 Poff<br>@27:15 P20, @27.45 Poff<br>@29.20 P20 en @ 30 minFurther 3x P20 and Poff, filling the vein with µPL and blood
Conclusion	The method to penetrate is difficult to establish. Predict.			

**March 10, 2014**	Location: Attempt: 27	Location: Attempt:28	Location: Attempt:29	Location: Attempt:
Eye #, right / left	TGpig68 OS			
User	TJ		MdS	
Start time /date /time in movie	18u00 10-3-2014	@5min		
Video name:<br>Fluor image name:	2014-3-10-18-12-31		2014-3-10-18-30-45	
Tool (RI, CS-thin, CS-thick)	Curved pipette 2			
Tool quality after surgery	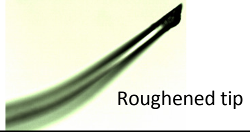			
Occlusion size	1500µm	1000µm (2e attempt)		
Occlusion location in eye	3800µm	@3500µm		
Vein diameter @ penetration	300µm			
Penetration location, relative to occlusion	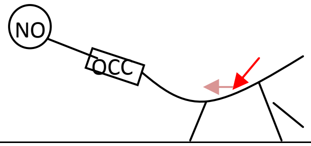	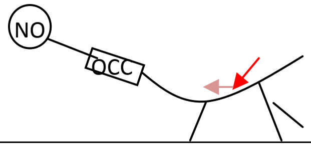	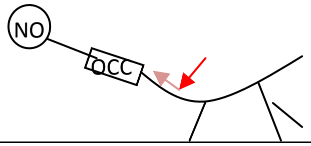	
Approaching angle				
Tool bevel orientation				
Number of piercing movements (µm)	Manual 1x	Manual	Manual	
Penetration				
Injection:(Intravitreal / flow visible in vein)				
Injection rate				
Total volume injected (µL)				
Occlusion resolved?<br>(n/a, yes, no, injection time)				
Hemmorrhage?		small		
Remarks<br>(if attempt cancelled, explain why)	The curved pipette is pushed slightly onto the vein, then only x and y is used to push the tip along the vein, while keeping pressure. Difficult procedure		The "curved pushing method" is difficult to perform on the left side with the right hand.	
